# Intestinal acetic acid regulates the synthesis of sex pheromones in captive giant pandas

**DOI:** 10.3389/fmicb.2023.1234676

**Published:** 2023-08-25

**Authors:** Ming-yue Zhang, Xiao-hui Zhang, Xue-ying Wang, Yu-liang Liu, Jun-hui An, Dong-hui Wang, Zhi-gang Cai, Rong Hou

**Affiliations:** ^1^Chengdu Research Base of Giant Panda Breeding, Chengdu, China; ^2^Sichuan Key Laboratory of Conservation Biology for Endangered Wildlife, Chengdu, China; ^3^Sichuan Academy of Giant Panda, Chengdu, China

**Keywords:** captive giant pandas, sex pheromone, mate preference, acetic acid, gut microbe

## Abstract

As a typical solitary animal, adult giant pandas rely on chemical signals (sex pheromones) to transmit reproductive information during oestrous. Although researchers have confirmed that the gut microbiota is related to the emission and reception of sex pheromones, there is no clear correlation between the gut microbes and the synthesis of sex pheromone of giant pandas, that is, which gut microbes and microbial metabolites are participate in the synthesis of giant panda’s sex pheromone. As a mirror of gut microbiota, fecal microbiota can reflect the composition of gut microbiota and its interaction with host to some extent. The purpose of this study is to explore how the gut microbes affect the synthesis of sex pheromones in captive giant pandas by combining analysis of the fecal microbiome and metabolomics. The results of correlation and microbial function analysis show that intestinal microorganisms such as *Veillonellaceae* and *Lactobacillilaceae* are associated with the synthesis of short chain fatty acid (acetic acid) and volatile ester metabolites, such as 1-butanol, 3-methyl, acetate, acetic acid, hexyl ester and 3-hexen-1-ol, acetate, (Z). In summary, based on this study, we believe that volatile metabolites such as fecal acetate participate in the process of mate preference of captive giant pandas and affect their expression of natural mating behavior. The possible mechanism is that the gut microbes can promote the synthesis of key chemical signaling substances in perianal glands through mediated intermediate fecal metabolites, thus affecting the normal information exchange between giant pandas individuals. The results of this study have greatly enriched our understanding of gut microbes regulating the synthesis of sex pheromones in giant pandas.

## Introduction

Intestinal microorganisms are important carriers for the interactions between the body and the environment. The neural network system of the intestine is also known as the “second brain.” The signals of the gastrointestinal tract can be transmitted to the brain through the brain-gut microbiome axis in the form of intestinal microorganism and tryptophan metabolism, thus affecting the physiological status and behavior of the host (Foster et al., 2017). The latest research results in Wu et al. (2021) show that specific gut microbes can influence social behavior through the discrete neuron circuit that mediated the stress response in the brain, and the social interaction between animals mediates basic behavior, including mating, breeding and defense. This shows that intestinal microorganisms not only affect the host’s metabolism and immune activities, but also profoundly affect the host’s behavior, including anxiety, cognition and social activities (Wu et al., 2021). Sharon et al. (2010) demonstrate the direct and subtle relationship between *Drosophila melanogaster* intestinal microflora and mating preference through exquisite experiments. The results showed that symbiotic bacteria (*Lactobacillus plantarum*) can influence mating preference by changing the levels of cuticular hydrocarbon sex pheromones (Sharon et al., 2010). In addition, Yi and Cha (2022) find that gut microbes also affects sexual attractiveness and mating preference in mammals that need to make mate preference through sex pheromones that determine individual scent. These results indicate that the gut microbes will affect the abundance of odour sex pheromones, thereby changing the mate preference of animals (Sharon et al., 2010; Yi and Cha, 2022).

The giant panda is a unique endangered relict species in China that is mainly distributed in the mountains of Sichuan, Shaanxi and Gansu provinces in China (Schaller et al., 1985). Mating choice is an important part of the mating process of giant pandas. Free mate choice is crucial to the protection of captive giant panda populations and the rejuvenation of small wild populations (Lumley et al., 2015; Martin-Wintle et al., 2015, 2018). Years of behavioral observation shows that both wild and captive giant pandas exhibit strong mate selection behavior (Martin-Wintle et al., 2015; Owen et al., 2016). During the short oestrous season in spring every year, wild giant pandas transmit their reproductive status through chemical signals and acoustics to attract mates and coordinate mating activities (Swaisgood et al., 2004; Charlton et al., 2010, 2011), while in other non-oestrous seasons, wild giant pandas rarely contact each other (in the wild environment, there are generally social behaviors of mothers and their cubs during the nursing period) (Schaller et al., 1985). As a typical solitary animal, the communication of giant pandas mostly depends on the transmission of sex pheromones during oestrous (Zhang et al., 2008). Although researchers have confirmed that giant pandas do have sex pheromones, which can transmit reproductive information (sex and oestrous status), it is not clear which sex pheromone component will affect the mating preference in male and female giant pandas. As a mirror of gut microbes, fecal microbiota can reflect the composition of gut microbiota and its interaction with host to some extent. Due to the difficulty in obtaining and sampling the intestinal tissue of giant pandas, feces have become the main source of research on gut microbiome and metabolites (Tang et al., 2020). The gut microbes may affect the mating preference of giant pandas for two main reasons. The first is to directly synthesize volatile metabolites under the action of intestinal microorganisms and functional enzymes, which may be the sex pheromone substances of giant pandas. Although academics believes that giant pandas transmit sex pheromones by using urine or perianal gland markers (Swaisgood et al., 2004), the possibility of faeces synthesizing sex pheromones also exists. Because, the perianal gland is a peripheral gland located near the anus, so when labeling the perianal gland in giant pandas, fecal microorganisms and their volatile metabolites may remain on the markers (trees), thereby affecting the communication of reproductive information in giant pandas. At the same time, some studies have also found that insects directly synthesize pheromones through intestinal microorganisms (Sharon et al., 2010), it has been reported that giant pandas may share the structure of sex pheromones with insects (Zhu et al., 2017); that is, their sex pheromones may be the same kind of substances that are synthesized by the intestinal or fecal microbes. On the other hand, the intestinal tract produces certain metabolic substrates and releases energy through the reaction of microorganisms and functional enzymes, which jointly regulate the synthesis of perianal gland chemical pheromones under the fermentation of the perianal gland microenvironment. Although some studies have confirmed that the synthesis of chemical signals in the perianal glands of giant pandas conforms to the “microbial fermentation hypothesis” (Theis et al., 2013; Zhou et al., 2021), the process requires the consumption of energy in the body and the corresponding substrate to be catalysed by functional enzymes to complete the synthesis of chemical signal substances. We all know that the main way the body obtains energy is from food. Under the action of the gut microbes, food is decomposed into carboxylic acids such as pyruvate, acetic acid, and lactic acid through glycolysis and under the action of enzymes, acetyl-CoA is synthesized to provide energy for the body (Comerford et al., 2014). Carboxylic acid is also an important substrate for the synthesis of sex pheromone esters and aldehydes (Schug et al., 2015). The latest study combining olfactory protein theory to reveal the composition of giant panda sex pheromones found that both long chain aldehydes and their corresponding carboxylic acids may be the main substances of giant panda sex pheromones (Zhu et al., 2017). Therefore, we speculate that carboxylic acid, an important metabolite of giant panda intestinal microbes, may be an intermediate substrate for the synthesis of sex pheromones in perianal glands.

Therefore, the purpose of this study is to preliminarily explore whether fecal microbes and microbial metabolites participate in the synthesis of sex pheromone and how they affect the mate preference and natural mating behavior expression of captive giant pandas by combining analysis of the intestinal microbiome and metabolomics. This research attempts to elucidate the chemical communication process of mate preference of giant pandas at the level of fecal microbiota, and reveal the mechanism that affects the decline in the natural reproduction behavior of captive giant pandas. The results of this study greatly enrich our understanding of the regulation of host mating behavior by intestinal microflora and provide a new theoretical basis for the use of gut or fecal microbes to regulate mating preference in the future.

## Materials and methods

### Sample collection

The collection of giant panda faeces in this experiment was carried out at the Chengdu Research Base of Giant Panda Breeding. Ten healthy adult giant pandas were selected and 24 faecal samples were collected in total. See Table 1 for sample information. The faecal samples were collected within 10 min after the giant panda defecated, and the outer layer of the faecal contact with the ground was stripped, the sample was put into a sterile bag, and the bag was discharged of air, put into an ice box and transported back to the laboratory and stored at - 80°C.

**Table 1:** Experimental grouping.

Groups	Name	Gender	Age	Sampling period	Sample count
NM	Gong Zai	Male	13	Jan, 2021- Feb, 2021	3
NM	Zhi Zhi	Female	12	Jan, 2021- Feb, 2021	3
NM	Mei Lan	Male	15	Jan, 2021- Feb, 2021	3
NM	Lou Abao	Male	14	Jan, 2021- Feb, 2021	3
AI	Ying Ying	Male	12	Jan, 2021- Feb, 2021	2
AI	Cheng Shuang	Male	9	Jan, 2021- Feb, 2021	2
AI	Ni Da	Female	6	Jan, 2021- Feb, 2021	2
AI	Cheng Da	Female	10	Jan, 2021- Feb, 2021	2
AI	Mei Lun	Female	8	Jan, 2021- Feb, 2021	2
AI	Mei Abao	Female	11	Jan, 2021-Feb, 2021	2

NM, During the mating process, the adult giant panda can express normal and complete natural mating behaviors, including courtship and mating behaviors; AI, During the mating process, the adult giant panda experience natural mating failure due to its inability to express normal courtship and mating behaviors, and insertion failure during mating is considered an abnormal natural mating behavior. For the definition of courtship and mating behavior, see our article on Applied Animal Behavior Science published in 2021 (Zhang et al., 2021).

The study was conducted according to the guidelines of the Chengdu Research Base of Giant Panda Breeding Institutional Animal Care and Use Committee (protocol code 2019012 and date of approval).

### Metagenomic sequencing of faecal microorganisms

#### Total DNA extraction and database construction

After collecting faecal samples, we immediately extracted microbial DNA, and all experimental steps were carried out in a biosafety cabinet. Because there was a large amount of undigested bamboo in the faeces of giant pandas, pretreatment was required before extracting the total DNA: approximately 10 g of faeces was placed in a 10 mL centrifuge tube, 8 mL of sterile PBS was added, and after 5 min of vortex oscillation, the microorganisms were separated from the particles in the faeces such as bamboo, and the supernatant was collected. After collecting the supernatant three times, the sample was centrifuged at 12000 × g for 10 min, and the microbial precipitate was collected and stored at - 70°C. DNA concentration was measured using Qubit^®^ dsDNA Assay Kit in a Qubit® 2.0 Fluorometer (Life Technologies, CA, United States). The OD value was between 1.8 ~ 2.0 and DNA contents above 1 μg were used to construct the library.

A total amount of 1 μg DNA per sample was used as input material for the DNA sample preparations. Sequencing libraries were generated using a NEBNext^®^ Ultra ™ DNA Library Prep Kit for Illumina (NEB, Ipswich, MA, United States), according to the instructions. Next, index codes were added to attribute sequences to each sample. Then, the DNA sample was fragmented by sonication to a size of 350 bp. Next, DNA fragments were end-polished, A-tailed, and ligated with the full-length adaptor for Illumina sequencing with further PCR amplification. PCR products were purified (AMPure XP system), and libraries were analysed for size distribution by an Agilent 2,100 Bioanalyzer and quantified using real-time PCR (Bio-Rad CFX96). A total of 35 cycles were set for the PCR test.

The clustering of the index-coded samples was performed on a cBot Cluster Generation System according to the manufacturer’s instructions. After cluster generation, the library preparations were sequenced on an Illumina NovaSeq platform, and paired-end reads were generated.

#### Information analysis of metagenomics

##### Sequencing results pretreatment

Preprocessing the raw data obtained from the Illumina sequencing platform using Readfq (V8) was conducted to acquire the clean data for subsequent analysis. The specific processing steps were as follows: (a) the reads that contained low-quality bases (default quality threshold value <38) above a certain portion (default length of 40 bp) were removed; (b) the reads in which the N base had reached a certain percentage (default length of 10 bp) were removed; (c) reads that shared the overlap above a certain portion were removed with Adapter (default length of 15 bp). Clean data were blasted to the host database, which defaults to using Bowtie2.2.4 software to filter the reads that were of host origin (Karlsson et al., 2013).

##### Metagenome assembly

(a) Single sample assembly: For the samples taken from noncomplex environments, such as intestines and faeces, the clean data were assembled and analysed by SOAP denovo software (V2.04) (Luo et al., 2012; Qin et al., 2014); then the assembled scaftigs were interrupted from the N connection, leaving the scaftigs without N (Nielsen et al., 2014). Clean Data from all samples were compared to each scaffold by Bowtie2.2.4 software to acquire the PE reads that were not used (Qin et al., 2014). (b) Mixed assembly: all the reads not used in the forwards step of all samples were combined, and then SOAPdenovo (V2.04)/MEGAHIT (v1.0.4-beta) software was used for mixed assembly with the same parameters as single assembly. The mixed assembled scaffolds were broken from the N connection, and obtained scaftigs were obtained. Fragment shorter than 500 bp in all scaftigs were filtered for statistical analysis both generated from single or mixed assembly.

##### Gene prediction and abundance analysis

(a) The scaftigs (> 500 bp) assembled from both single and mixed samples all predicted the ORF by MetaGeneMark (V2.10) software, and length information shorter than 100 nt was filtered from the predicted result with default parameters (Qin et al., 2014). (b) For ORF prediction, CD-HIT software (V4.5.8) was adopted for redundancy and to obtain the unique initial gene catalogue (the genes here refer to the nucleotide sequences coded by unique and continuous genes) (Fu et al., 2012; Sunagawa et al., 2015). (c) The clean data of each sample were mapped to the initial gene catalogue using Bowtie2.2.4, and the number of reads to which genes mapped in each sample was obtained (Qin et al., 2014). The genes with <2 in each sample were filtered, and the gene catalogue (unigenes) was eventually used for subsequent analysis (Qin et al., 2012). (d) Based on the number of mapped reads and the length of the gene, the abundance information of each gene in each sample was statistically analysed. (e) The basic information statistics, core-pan gene analysis, correlation analysis of samples and Venn figure analysis of the number of genes were all based on the abundance of each gene in each sample in the gene catalogue.

##### Taxonomy annotation

(a) DIAMOND software (V0.9.9) was used to blast the unigenes to the sequences of Bacteria, Fungi, Archaea and Viruses, which were all extracted from the NR database (Version: 2018-01-02) of NCBI with the parameter setting blastp -e 1e-5 (Buchfink et al., 2015). (b) For the finally aligned results of each sequence, as each sequence might have multiple aligned results, we chose the result of which the e value≤1e-5 to take the LCA algorithm which was applied to system classification of MEGAN software to ensure the species annotation information of sequences (Huson et al., 2011; Oh et al., 2014). (c) The table containing the number of genes and the abundance information of each sample in each taxonomic hierarchy (kingdom, phylum, class, order, family, genus and species) were obtained based on the LCA annotation result and the gene abundance table. The abundance of a species in one sample equalled the sum of the gene abundance annotated for the species; the gene number of species in a sample equalled the number of genes whose abundance was nonzero. (d) Krona analysis, the exhibition of generation situation of relative abundance, the exhibition of abundance cluster heatmap, principal coordinate analysis (PCoA) (R ade4 package, Version 2.15.3) and nonmetric multidimensional scaling (NMDS) (R vegan package, Version 2.15.3) decrease-dimension analysis were based on the abundance table of each taxonomic hierarchy. PCoA revealed the clustering pattern based on mating preference. Bray-Curtis was used to plot this PCoA. The difference between groups was tested by ANOSIM analysis (R vegan package, Version 2.15.3) (Avershina et al., 2013; Rivas et al., 2013). Meta stats and LEfSe analysis were used to look for the different species between groups. A permutation test between groups was used in metastasis analysis for each taxonomy, and the *p* value was obtained. Then the Benjamini and Hochberg false discovery rate was used to correct the p value and acquire the q value. LEfSe analysis was conducted by LEfSe software (the default LDA score was 2) (White et al., 2009; Segata et al., 2011).

##### Common functional database annotations

(a) DIAMOND software (V0.9.9) was adapted to blast unigenes to the functional database with the parameter setting of blastp, −e 1e-5 (Avershina et al., 2013). To clarify the functions of microorganisms, we annotated the functions of protein-coding genes identified in the whole metagenomics data according to the metabolic pathway (KEGG) homologous gene cluster (eggNOG) and carbohydrate enzyme (CAZy) (Cantarel et al., 2009; Kanehisa et al., 2014; Powell et al., 2014). For each sequence’s blast result, the best Blast hit was used for subsequent analysis (Feng et al., 2015). (b) Statistics of the relative abundance of different functional hierarchies. The relative abundance of each functional hierarchy equals the sum of the relative abundance annotated to that functional level. (c) Based on the functional annotation results and gene abundance table, the gene number table of each sample in each taxonomic hierarchy was obtained. The gene number of a function in a sample equals the gene number annotated to this function and the abundance is nonzero. (d) Based on the abundance table of each taxonomic hierarchy, not only the counting of annotated gene numbers, the exhibition of the general relative abundance situation, the exhibition of abundance cluster heatmap and the decrease-dimension analysis of PCoA and NMDS were conducted, but also the ANOSIM analysis of the difference between groups (inside) based on functional abundance, comparative analysis of metabolic pathways, the Metastats and LEfSe analysis of functional difference between groups were performed.

### Untargeted metabolomics analysis based on Gas chromatography–mass spectrometry (GC–MS)

Furthermore, for metagenomics research of intestinal microorganisms, we conducted an untargeted metabolomics analysis of samples based on GC–MS.

#### Metabolite extraction

First, an appropriate amount of sample was placed in a 20 mL headspace vial, and 4 mL of saturated sodium chloride aqueous solution was added to it; then, 10 μL of the ISTD solution was added to each sample; incubate the samples were then incubated at 60°C for 10 min; the SPME fiber was placed in the chamber at 270°C for 10 min before extraction; the SPME was transferred to the incubator at 60°C for 40 min; and the SPME fibre was desorbed at 250°C for 5 min in a GC injector. Finally, the SPME fibre was placed in the chamber at 270°C for 10 min after the injection step.

#### GC–MS analysis

Analyses were carried out using a LECO Pegasus^®^ 4D instrument (LECO, St. Joseph, MI, United States) consisting of an Agilent 8890A GC (Agilent Technologies, Palo Alto, CA, United States) system equipped with a split/splitless injector, and dual stage cryogenic modulator (LECO) coupled with TOF-MS detector (LECO). An Rxi-5Sil MS column (30 m × 250 μm I.D., 0.5 μm) (Restek, United States) was used as the first dimension column (1 D), and an Rxi-17Sil MS column (2.0 m × 150 μm I.D., 0.15 μm) (Restek, United States) was used as the second-dimension column (2 D). The carrier gas was high purity helium (>99.999%) at a constant flow rate of 1.0 mL/min. The temperature program of the oven was as follows: the oven temperature was held at 50°C for 1 min at first, then raised to170 °C at a rate of 2°C /min and held for 1 min, then raised to 230°C at a rate of 30°C /min and held for 1 min. The secondary oven temperature was 5°C higher than the first oven. The temperature of the modulator was always 3°C higher than that of the second column. The modulator was operated with a 0 s modulation period. The GC injector temperature was 250°C.

Flavour substance analysis was performed with LECO Pegasus BT 4D. The transfer line and TOFMS ion source temperatures were set at 250°C and 250°C, respectively. The acquisition frequency was 20 spectra/s. The mass spectrometer was operated in EI mode at 70 eV using a range of m/z 35–550 and a detector voltage of 1984 V (Boukaew et al., 2021).

#### Data preprocessing

Raw data analysis, including peak extraction, baseline adjustment, deconvolution, alignment and integration, was completed with Chroma TOF (V4.72.0.0) software and the NIST2017 database was used for metabolite identification by matching the mass spectrum and retention index.

### Correlation analysis

The Spearman statistical method was used to analyse the correlation coefficient between the significantly different microbial communities and significantly different metabolites screened in experimental samples, and R language and Cytoscape software were combined for matrix heatmap analysis to explore the interaction relationship between microbial communities and metabolites.

## Results

### Effects of mating preference on fecal microbiota in adult giant pandas

#### Species alpha and beta diversity analysis

**Figure 1:**
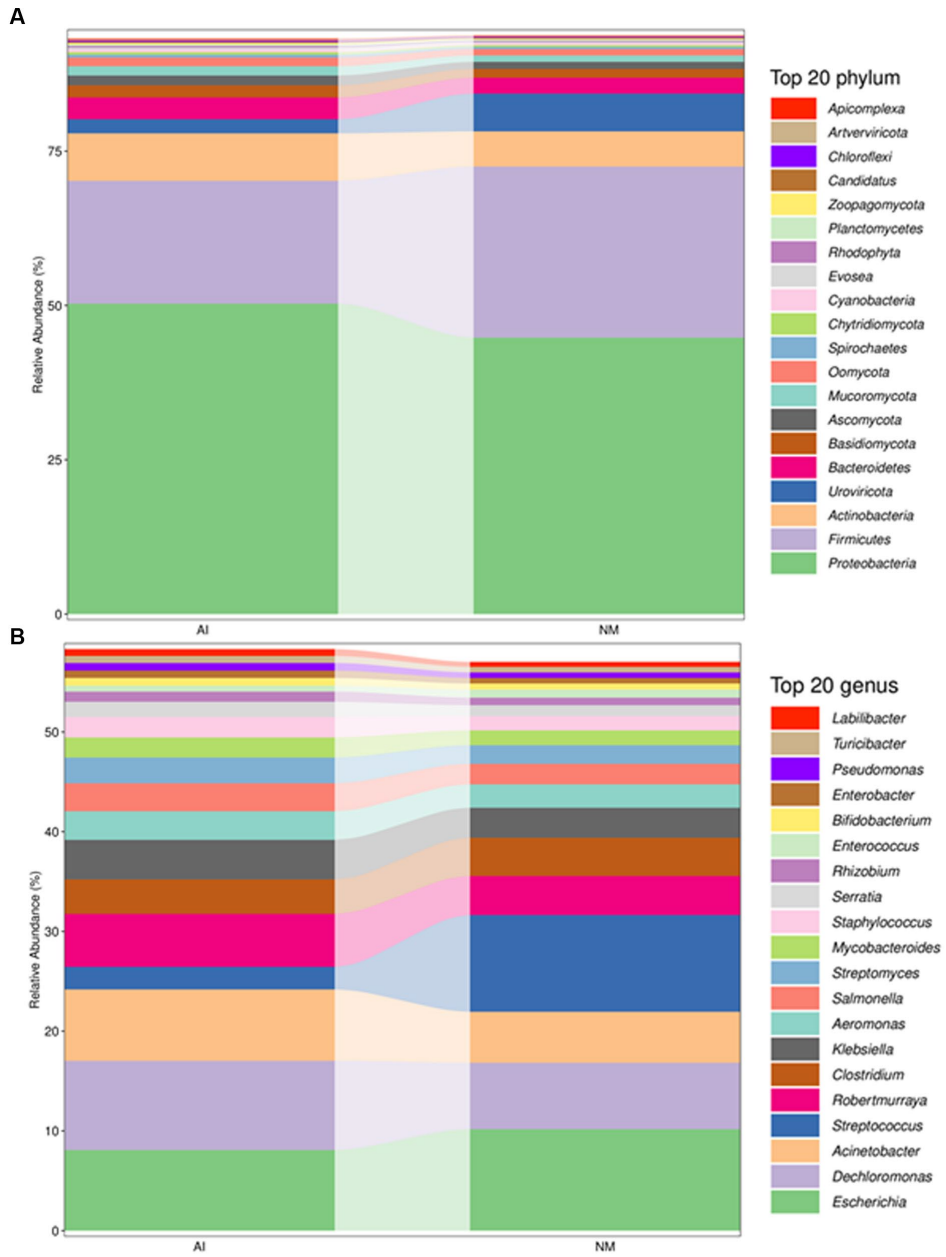
Column chart of relative abundance at the phylum **(A)** and genus **(B)** levels in the faecal microbiota of giant pandas between NM and AI groups. In this figure, the abscissa is arranged according to the sample name, each bar represents a sample, each taxon is distinguished by colour, and the ordinate represents the relative abundance of each taxon, the longer the bar is, the higher the relative abundance.

Through metagenomics sequencing and species annotation, a total of 36 bacterial phyla and 139 bacterial genera were identified from the faeces of 10 adult giant pandas. The most abundant phylum was *Proteobacteria* (Figure 1A), accounting for 47.5% of the total bacterial colonies, and the most abundant genus was *Escherichia* (Figure 1B).

Supplementary Table S1 showed that for the fecal biological community of giant pandas in the NM group, the Simpson, Chao1 and Shannon diversity indices were higher than those of giant pandas in the AI group (Supplementary Table S1), but the difference was not significant (*p* > 0.05), which indicated that the abundance and diversity of the fecal biota of giant pandas in the NM group were higher than those of giant pandas in the AI group.

Supplementary Figure S1 showed that the fecal microbial community structures of giant pandas in the NM group and AI group were similar. It could be seen from the results in Supplementary Tables S2, S3 that the β diversity index of the fecal microbes of giant pandas in each group (Adonis and ANOSIM) was not significantly different (*p* > 0.05) (Supplementary Tables S2, S3).

#### Analysis of taxonomic differences and functional differences

**Figure 2:**
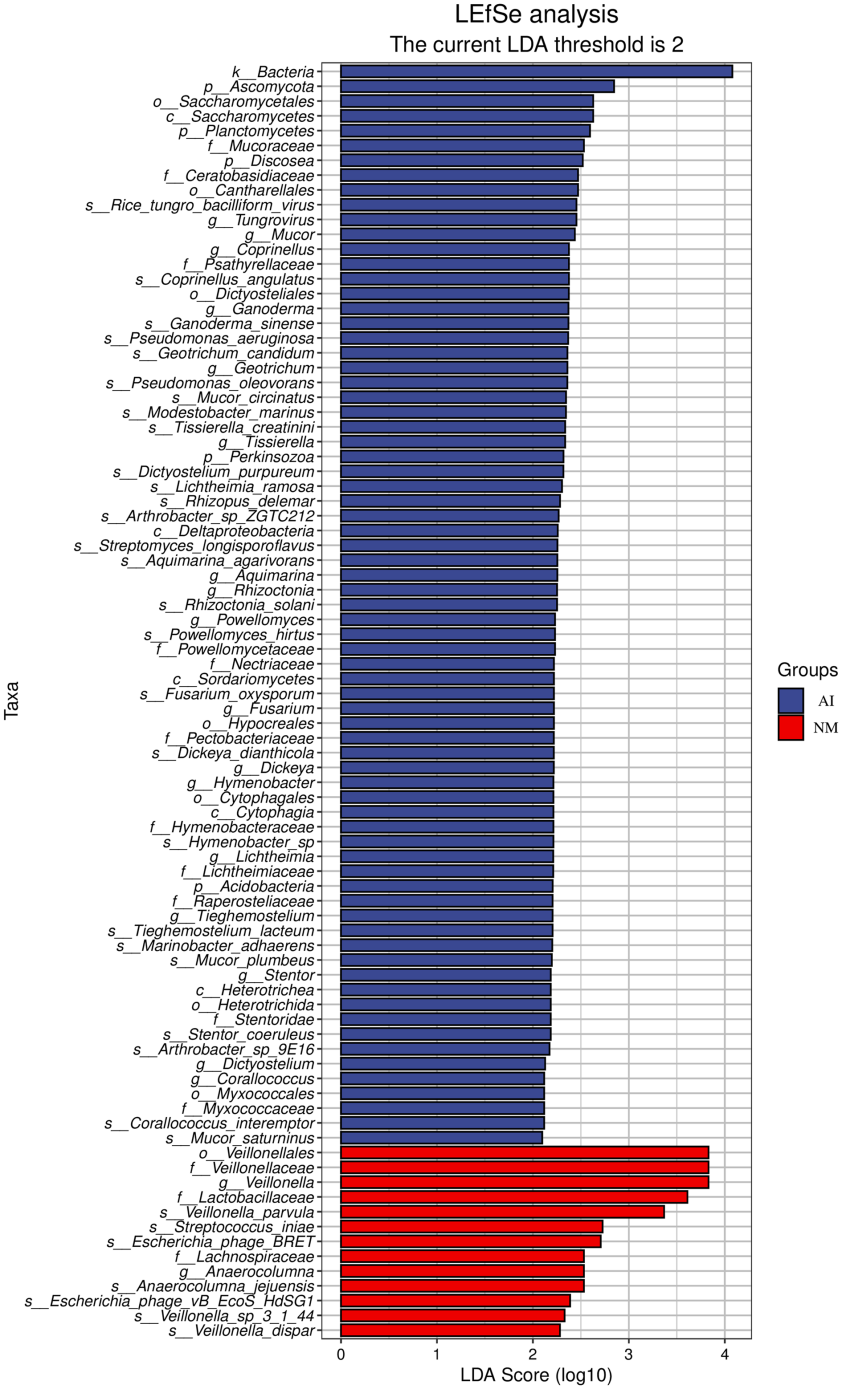
LEfSe species histogram of gut microbes. In the figure, the ordinate is the taxon with significant differences between groups, and the abscissa is the Bar chart to visually display the LDA analysis log score of each taxon. The classification units are sorted according to their score values to describe their specificity in sample grouping. The longer the length is, the more significant the difference of the taxon is. The color of the Bar chart indicates the sample group with the highest abundance corresponding to the taxon.

Although the β diversity difference between the two groups was not significant, there were still species with significant differences in relative abundance (*p* < 0.05). According to the results of LEfSe analysis, in the NM group, the most significant difference was found in the families *Veillonellaceae* and *Lactobacillus* and in the genus *Veillonella*. Among them, *Veillonella parvula* and *Veillonella dispar* strains under *Veillonella* were also significantly upregulated in the NM group. In the AI group, 17 species of genera such as *Tungrovirus*, *Mucor*, and *Coprinellus* significantly increased (Figure 2).

**Figure 3:**
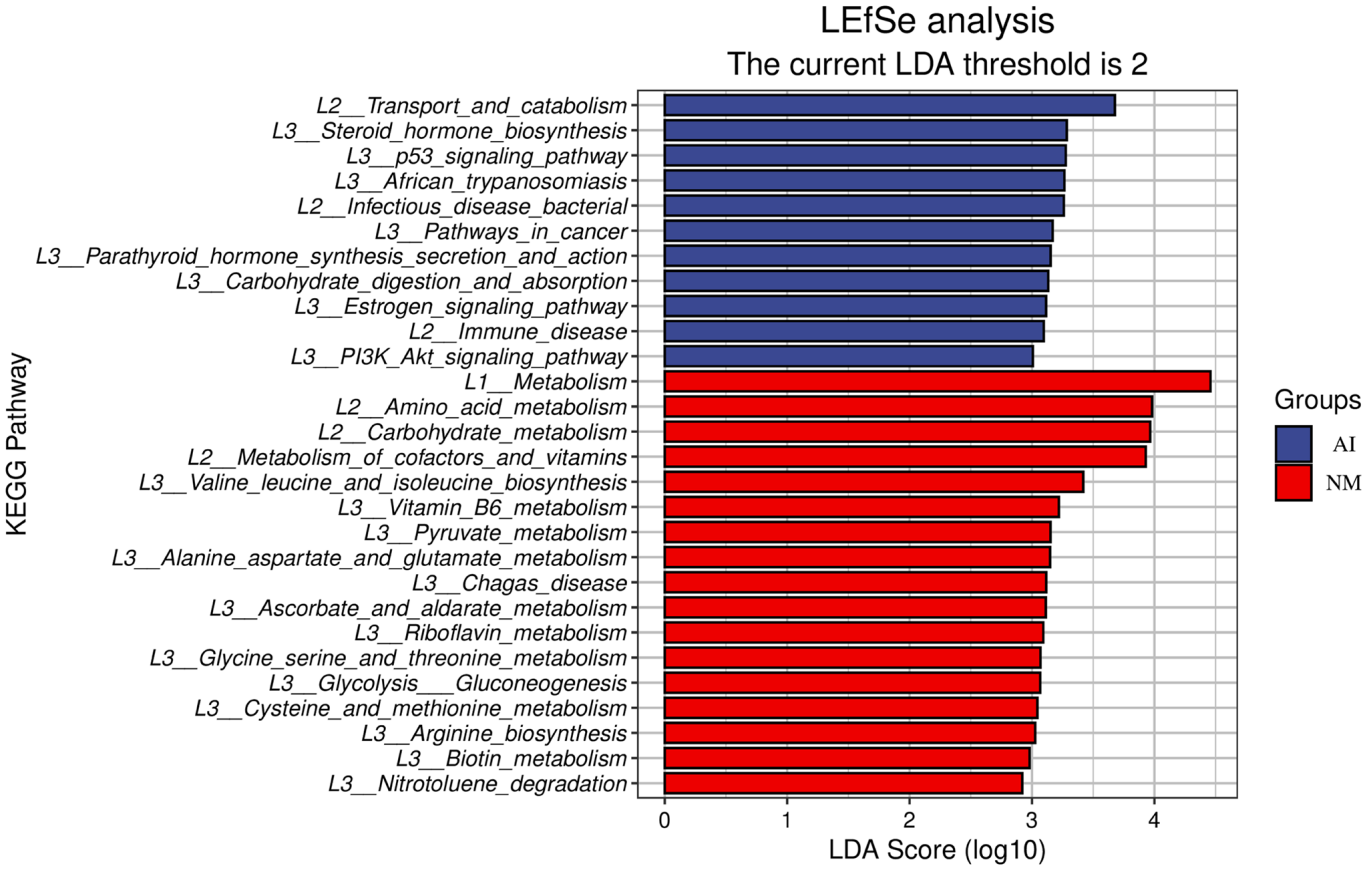
LEfSe functional histogram. In this figure, the ordinate is the taxa with significant differences between groups, and the abscissa is a bar chart to visually display the LDA analysis logarithmic score value of each taxon. The taxa are ordered by the size of the score value to describe their specificity in the sample grouping. The longer the length, the more significant the difference of the taxon is, and the colour of the bar chart indicates the most abundant sample grouping corresponding to the taxon.

The comparative analysis of the microbial metabolic spectrum showed that the amino acid metabolism and carbohydrate metabolism function genes in the NM group were significantly increased compared with those in the AI group (Figure 3). Furthermore, through LEfSe analysis, it was found that there were three carbohydrate metabolic pathways and five amino acid metabolic pathways with significant differences between the NM and AI groups. The three carbohydrate metabolic pathways included ascorbate_ and_ aldarate_ metabolism (ko00053) (LDA score: 3.1129; *p* < 0.05), glycolysis_ gluconeogenesis (ko00010) (LDA score: 3.0675; *p* < 0.05) and pyruvate_ metabolism (ko00620) (LDA score: 3.1513; *p* < 0.05) metabolic pathways, five amino acid metabolism pathways including alanine_ aspartate_ and_ glutamate_ metabolism (ko00250) (LDA score: 3.1479; *p* < 0.05), arginine_ biosynthesis (ko00220) (LDA score: 3.0267; *p* < 0.05), cysteine_ and_ methionine_ metabolism (ko00270) (LDA score: 3.0438; *p* < 0.05), glycine_ serine_ and_ threonine_ metabolism (ko00260) (LDA score: 3.0696; *p* < 0.01) and valine_ leucine_ and_ isoleucine_ biosynthesis (ko00290) (LDA score: 3.4191; *p* < 0.05) metabolic pathways (Figure 3).

Furthermore, the annotation results of functional enzymes related to the metabolism of these 5 amino acids and 3 carbohydrate metabolism pathways showed significant differences between the two groups, such as enzymes related to pyruvate metabolism, such as pyruvate oxidase [EC 1.2.3.3], pyruvate formate lyase [EC 2.3.1.54], acetyl-CoA synthetase [EC: 6.2.1.1], acetic acid kinase [EC 2.7.1.1], acyl phosphatase-1 [EC 3.6.1.7], and 2-ketoate oxidoreductase [EC 1.2.7.11] (Supplementary Figures S2, S5–S20).

### Effects of mating preference on fecal metabolites in adult giant pandas

#### Differential metabolite analysis

Based on the method of univariate statistics, differential metabolites with FC > 1.5 or FC < 0.67 and *p* value<0.05 were visualized in the form of volcanic maps, and the significance of metabolic changes between the two group samples was visually shown (Supplementary Figure S3). Univariate analysis visually showed the significance of metabolite changes between the two groups of samples. Then, we used multivariate statistics to evaluate the partial least squares discrimination analysis (PLS-DA) obtained by 7-fold cross validation. The evaluation results showed that the model was stable, the original model did not have overfitting, and the model was robust (Supplementary Figure S4).

#### Bioinformatics analysis of differential metabolites

The results of GC–MS analysis showed that, compared with the NM group, 33 volatile compounds in the feces of captive giant pandas in the AI group underwent significant changes, mainly to metabolic substances such as fatty acids, alcohols, esters, and ketones. Among them, according to the strict OPLS-DA VIP > 1 and *p* value<0.05 as significant differential metabolite screening criteria, 9 species showed significant upregulation and 24 species showed significant downregulation (Supplementary Figure S3). Our results indicated that the relative abundance of volatile alcohols and esters, as well as short-chain fatty acid (acetic acid), such as 1-hexanol; acetic acid hexyl ester and 3,3-dimethylglutaric acid in the faeces of giant pandas in the NM group was significantly higher than that in the AI group, while the expression levels of ketones, ethers, and alkanes, such as 3-octanone, ethyl ether were significantly lower in the AI group (Supplementary Table S4).

**Figure 4:**
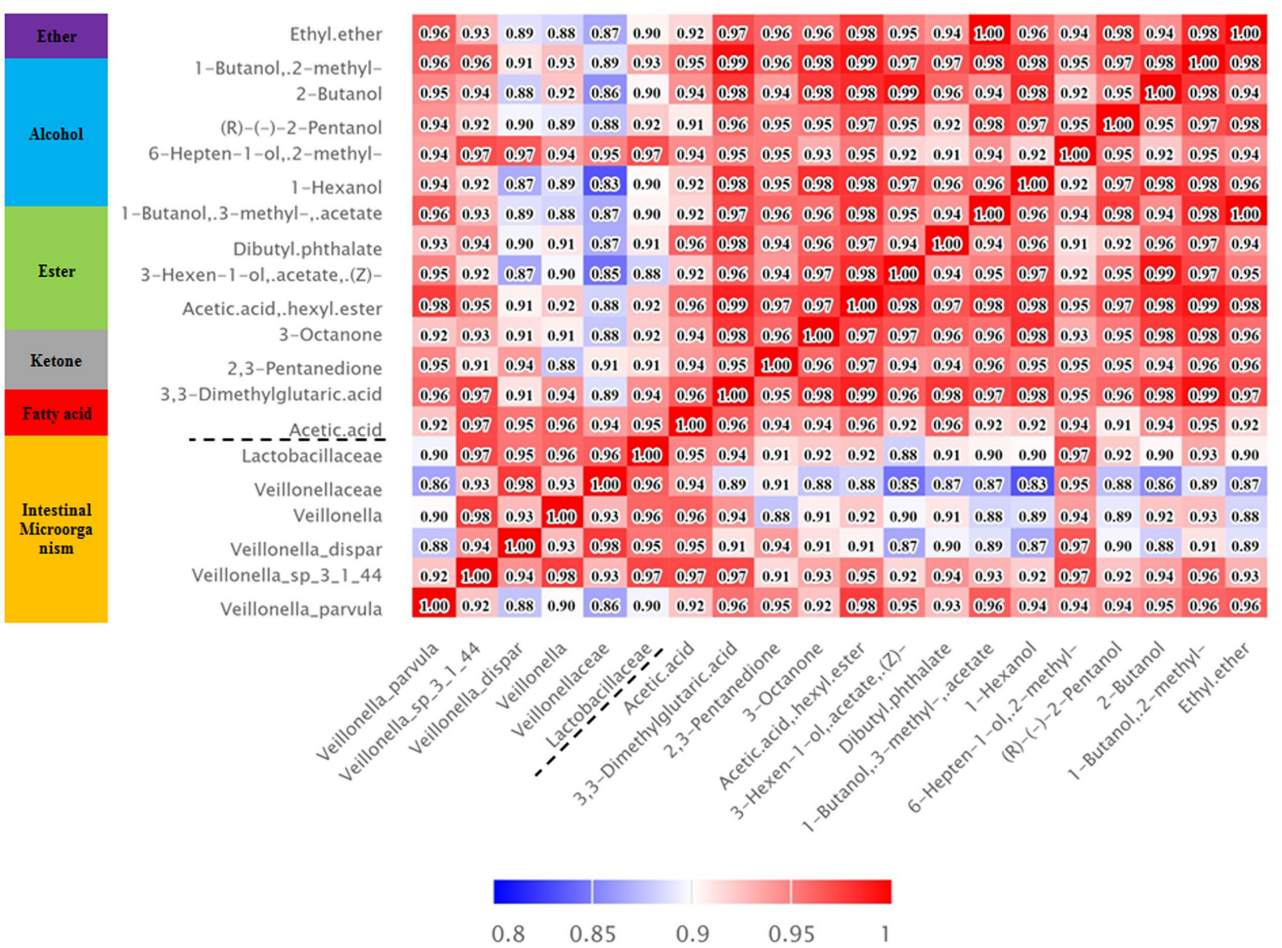
Heatmap of the correlation between significantly different intestinal microorganisms and metabolites. Red indicates a positive correlation, blue indicates a negative correlation, and white indicates a nonsignificant correlation. The colour depth is related to the absolute value of the correlation coefficient, that is, the higher the degree of positive or negative correlation, the darker the colour.

**Figure 5:**
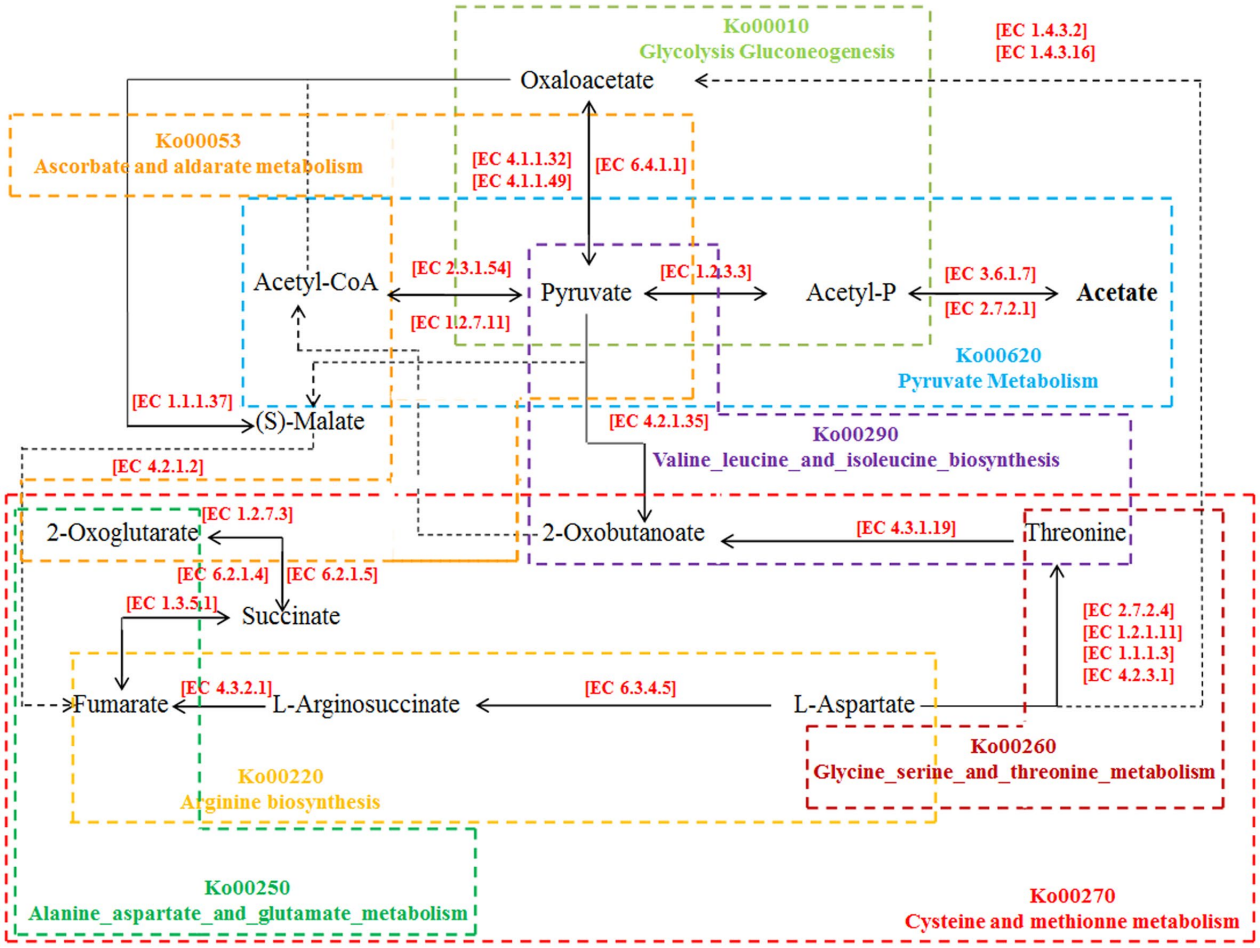
Pathways of influence of intestinal microorganisms on acetate synthesis in giant pandas. All KEGG Orthology numbers (KO) and Enzyme numbers (EC) were obtained from the KEGG database.

The correlation analysis results also showed that the metabolic substances of intestinal esters (acetic acid hexyl ester and 3-hexen-1-ol, acetate, (Z) -) showed a significant positive correlation with volatile alcohols such as 1-hexanol and (R) - (−) -2-pentanol, while acetic acid showed a significant positive correlation with fecal microbes such as *Veillonellaceae* and *Lactobacillilaceae* (Figure 4). According to the analysis results of the inter group differences of the functional unit Kegg Orthology (KO) of the fecal microbes and the predicted KEGG metabolic pathway (Supplementary Figures S5–S20), we preliminarily inferred that fecal microbes and functional enzymes affected the pathways of acetate synthesis (Figure 5). These results suggested that the important volatile metabolites of giant panda fecal microbes might be involved in the synthesis of chemical signals of gonad sex pheromones.

## Discussion

Previous studies have compared the composition of odorants and symbiotic flora in the perianal glands and faeces of giant pandas, and found that not only is the composition of odorants significantly different but their symbiotic flora is also significantly different at phylum and genus levels (Zhou et al., 2021). This shows that in the process of synthesis of chemical signals from the giant panda perianal gland, the fecal microbes and perianal gland flora have different functional divisions. In fact, perianal gland microbes are not a simple extension of intestinal microbes and there may be a unique microbial community in the anal genital gland that participates in the synthesis of chemical signals together with intestinal microorganisms and metabolites (Zhou et al., 2021). In this experiment, we analysed the results of volatile metabolites in the faeces of giant pandas using GC–MS. We found that volatile ester fecal metabolites synthesized by gut microbes, such as 1-butanol; 3-methyl -, acetate; acetic acid, hexyl ester and 3-hexen-1-ol, acetate, (Z) -, significantly promote the mating preference in giant pandas. At the same time, from the results of microbiology and correlation analysis, it was also found that the fecal microorganisms *Veillonella* and *Lactobacillaceae* were associated with the synthesis of these volatile substances. Extensive observational evidence indicates that pandas do not use faeces for chemical communication (Schaller et al., 1985; Pan et al., 2001). Therefore, we speculate that the formation of these metabolites by the fecal microbes is highly likely to play a crucial role in the synthesis of chemical signals in the perianal glands of giant pandas.

Acetic acid can be synthesized in two ways in the intestine: pyruvate generates acetic acid under the action of pyruvate oxidase [EC 1.2.3.3]; pyruvate first produces acetyl-CoA by the action of pyruvate dehydrogenase [EC 1.2.5.1] and then forms acetic acid by the catalysis of phosphotransacetylase [EC 2.7.2.12] and acetic acid kinase [EC 2.7.2.1] (Dalile et al., 2019). In this process, pyruvate plays an important role in the middle. Based on the analysis results of giant panda faecal metagenomics and amino acid-targeted metabolomics, it was found that the fecal microorganism *Veillonella* and its important short-chain fatty acid metabolite acetic acid and related enzymes in the faeces of giant pandas in the NM group were significantly upregulated, indicating that acetic acid may be the main substance affecting the mating preference of captive giant pandas. According to the analysis results of the differences between groups of functional unit KO of fecal microbes and the predicted KEGG metabolic pathway, we found that the expression of acetate affecting the mating preference of giant pandas may involve a variety of amino acid and carbohydrate metabolic pathways, among which pyruvate metabolism (ko00620) plays the most important role (Byron and Lindsay, 2017). Oxaloacetate produced by the glycolysis- gluconeogenesis pathway (ko00010) is an important complement pathway of the tricarboxylic acid cycle. It is connected to the pyruvate metabolism pathway through pyruvate carboxylase [EC 6.4.1.1] and to the ascorbate and aldarate metabolism pathway (ko00053) through malate dehydrogenase [EC 1.1.1.37] (Byron and Lindsay, 2017). In addition, through the results of this experiment, we also found that in addition to the carbohydrate metabolism-related pathways, alanine aspartate and glutamate metabolism (ko00250), arginine biosynthesis (ko00220), cysteine and methionine metabolism (ko00270), glycine serine and threonine metabolism (ko00260) and valine leucine and isoleucine biosynthesis (ko00290) and other amino acid metabolic pathways also participate in the synthesis of acetate. They are connected by key metabolites such as 2-oxoglutarate, are finally metabolized to acetyl-CoA or pyruvate under the catalysis of related enzymes, and participate in the metabolism of pyruvate to finally form acetic acid (Mehra et al., 2020) (Supplementary Figures S5–S20). In addition, we also found that the content of a metabolite (ether) that plays an important role in acetic acid synthesis in the faeces of the NM group of giant pandas was significantly lower than that of the AI group of giant pandas, indicating that the source of fecal acetic acid may also be ether entering the anal canal through the rectum and coming into contact with the natural air environment.

Previous research results show that several volatile fatty acids and aldehydes exist in the secretion of the giant panda’s perianal gland during oestrous, but it is not clear which volatiles may be the key pheromones affecting their mate selection (Hagey and MacDonald, 2003; Zhang et al., 2008). In the analysis of fecal volatile metabolites, we found that the significant increase in the abundance of volatile acetate substances affected the expression of mate preference and natural mating behavior of captive giant pandas, which indicated that fecal volatile metabolites synthesized by the interaction of gut microbiota and host might be the main sex pheromone component of captive giant pandas, and verified our first hypothesis that gut microbiota affected the synthesis of sex pheromone in giant pandas. Additionally, Zhu et al. (2017) obtain potential giant panda sex pheromones by screening the ligands of odorant-binding proteins (OBPs), whose structure is similar to that of moth sex pheromone components (Zhu et al., 2017). The main component of moth’s sex pheromone is dodecenyl acetate, which seems to indicate that acetate may be a substance potentially affecting the synthesis of giant panda’s sex pheromone (Rasmussen et al., 1996). In this experiment, the samples collected during differential expression of natural mating behaviors of giant pandas revealed a significant increase/decrease in acetic acid and 1-hexanol alcohols. Based on previous speculations, the results of this experiment further confirm that fecal metabolites such as acetic acid and 1-hexanol synthesized by gut microbes are important intermediate substances that mediate the fermentation and synthesis of chemical signals by perianal gland microbes (Zhou et al., 2001). In other words, the key chemical pheromone substances that affect the mating preference of giant pandas are most likely based on acetic acid and volatile alcohols as substrates, and the volatile substances formed by microbial enzymes in perianal glands are most likely acetate substances, which is consistent with the speculation of Zhu et al. (2017) that giant pandas and moths may share the acetate substance component of sex pheromone with a similar structure by using the method of reverse chemical ecology (Zhu et al., 2017). The results of this experiment further verify our second hypothesis that gut microbiota affects the synthesis of sex pheromone of captive giant pandas, and explain the mechanism of fecal metabolites affecting sex pheromone, but whether this substance is truly a key pheromone affecting the normal expression of natural mating behavior of giant pandas still needs much follow-up work to verify.

In this study, although we find that fecal acetic acid has an impact on the mating preference of captive giant pandas during breeding seasons, which may be related to the synthesis of sex pheromones, how do captive giant pandas use acetate to produce sex pheromone signals? Several enzymes are involved in the conversion of acetate into compounds such as dodecyl acetate. A key and potentially rate-limiting enzyme in this biosynthetic pathway is acyl-CoA short chain synthetase family member 2 (Acss2), which converts acetate and coenzyme A into acetyl-CoA for use in lipid synthesis (Luong et al., 2000; Xu et al., 2018). Our research find that the acetyl-CoA synthetase [EC: 6.2.1.1] in the Acss2 family is significantly upregulated in the NM group (Supplementary Figure S5). Therefore, we suspect that giant pandas use acetyl-CoA synthetase [EC: 6.2.1.1] to convert intestinal acetic acid into acetyl coenzyme A, providing energy and important substrates for genital gland organs such as perianal glands to synthesize odour pheromones.

## Conclusion

**Figure 6:**
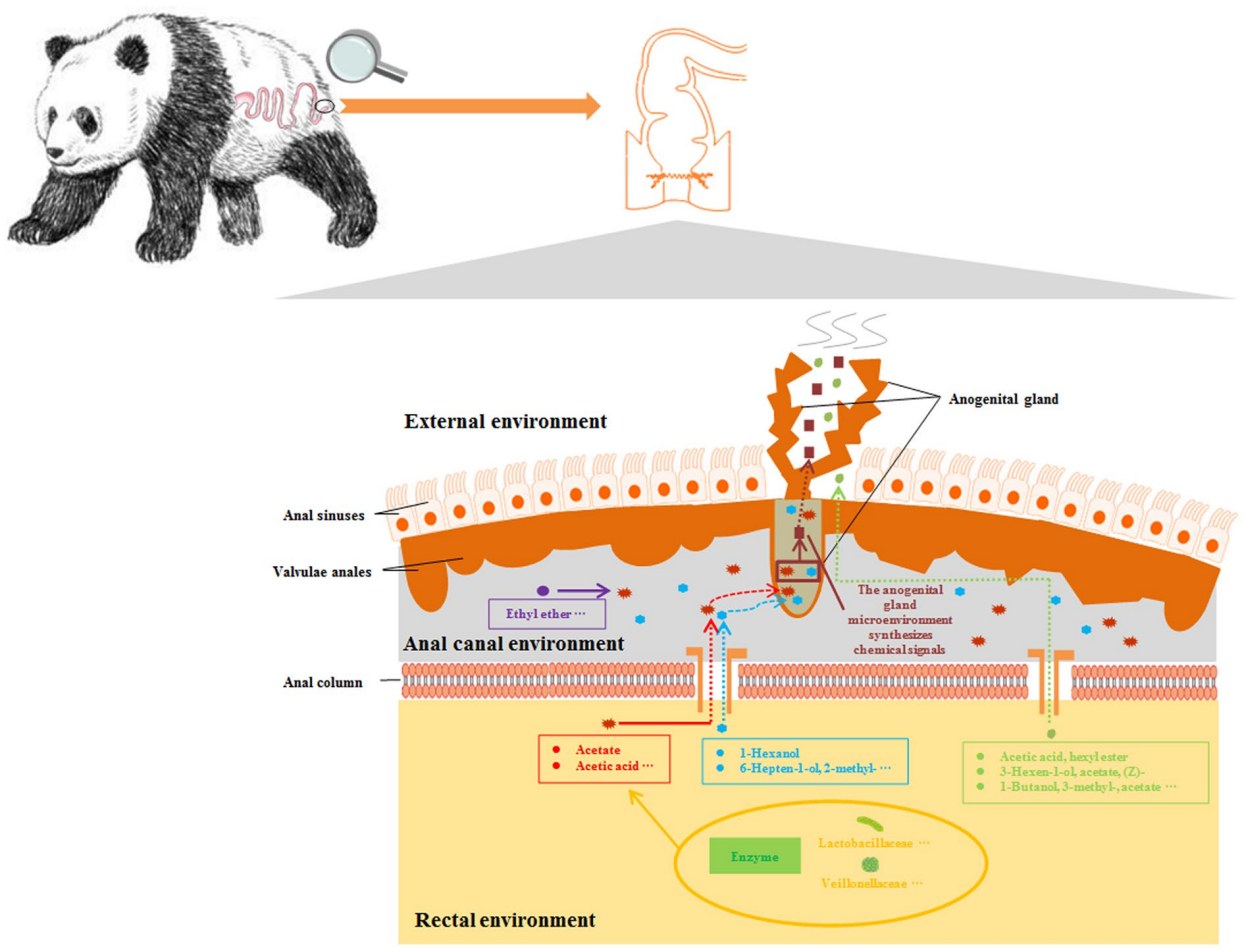
Reconstruction of the pathway of intestinal microorganisms participating in the synthesis of sex pheromones in captive giant pandas. Microbial metabolites in the intestine produce odours or mediate the synthesis of odours by the perianal glands, which may be used as chemical signals in the environment.

In summary, based on the results of this study, we believe that volatile metabolites such as fecal acetate participate in the process of mate preference of captive giant pandas and affect their expression of natural mating behavior. The possible mechanism is that the gut microbes can promote the synthesis of key chemical signaling substances in perianal glands through mediated intermediate fecal metabolites, thus affecting the normal information exchange between giant pandas individuals (Figure 6). In the future, we will need to combine functional analysis of the association between perianal glands and gut microbiota, as well as conduct further behavioral validation experiments, to identify key information components that affect mate preference and natural mating behavior expression in captive giant pandas. The results of this study have greatly enriched our understanding of gut microbes regulating the synthesis of sex pheromones in giant pandas, providing a theoretical basis for scientifically guiding the conservation and management of captive giant panda populations through the reconstruction of specific microbial or fecal communities.

## Data availability statement

The datasets presented in this study can be found in online repositories. The names of the repository/repositories and accession number(s) can be found in the article/Supplementary material.

## Ethics statement

The animal study was approved by Chengdu Research Base of Giant Panda Breeding Institutional Animal Care and Use Committee (protocol code 2019012 and date of approval). The study was conducted in accordance with the local legislation and institutional requirements.

## Author contributions

M-yZ, Y-lL, and RH conceived the ideas and designed methods. X-hZ, X-yW, Z-gC, J-hA, and D-hW collected the samples and conducted experiments. X-yW and M-yZ analyzed data. M-yZ and X-hZ wrote the manuscript. All authors contributed to the article and approved the submitted version.

## Funding

This study was supported by National Natural Science Foundation of China (32100386) and the Program of the Chengdu Research Base of Giant Panda Breeding (2020CPB-B05 and 2022CPB-P05).

## Conflict of interest

The authors declare that the research was conducted in the absence of any commercial or financial relationships that could be construed as a potential conflict of interest.

## Publisher’s note

All claims expressed in this article are solely those of the authors and do not necessarily represent those of their affiliated organizations, or those of the publisher, the editors and the reviewers. Any product that may be evaluated in this article, or claim that may be made by its manufacturer, is not guaranteed or endorsed by the publisher.
