# Supplementary material for: Intestinal acetic acid regulates the synthesis of sex pheromones in captive giant pandas
Source: Front Microbiol. 2023 Aug 25;14:1234676. doi: 10.3389/fmicb.2023.1234676 (PMC10485365; doi:10.3389/fmicb.2023.1234676)
Supplement: Supplementary file 1 [file Data_Sheet_1.docx]

**TABLE S1 α diversity index of gut microbes of giant pandas between the NM and AI groups**

| **Sample** | **Simpson** | **Chao1** | **Shannon** |
| --- | --- | --- | --- |
| **NM** | 0.9693 ± 0.0024 | 5053.2500 ± 247.8586 | 7.0620 ± 0.0887 |
| **AI** | 0.9638 ± 0.0041 | 4141.9167 ± 407.2317 | 6.7083 ± 0.1767 |
| ***p*-value** | 0.259 | 0.069 | 0.087 |

**Note:** The first column in the table is the sample name, and the following three columns are for the NM and AI groups, respectively α diversity index results of Simpson, Chao1 and Shannon (mean ± S.E.); The P-value reflects the significant difference in the α diversity index between the two groups; P<0.05 indicates a significant difference, and P<0.01 is the most significant difference.

**TABLE S2 Adonis species composition difference analysis results between groups**

| **items** | **Df** | **SumsOfSqs** | **MeanSqs** | **F.Model** | **R2** | **Pr(＞F)** |
| --- | --- | --- | --- | --- | --- | --- |
| Treat2 | 1 | 0.22548 | 0.22548 | 2.1987 | 0.09086 | 0.121 |
| Residuals | 22 | 2.25613 | 0.10255 |  | 0.90914 |  |
| Total | 23 | 2.48160 |  |  | 1.00000 |  |

**Note:** In this table, "Df" represents the degree of freedom, "SumsOfSqs" and "MeanSqs" represent the sum of squares of deviations and the mean square deviation respectively, "F.Model" represents the test value of F statistics, and "R2" represents the proportion of grouping variance and residuals to the total variance, that is, the proportion of differences in the original data that can be explained by grouping. "Pr (>F)" is the P value obtained by the displacement test. Generally, focus on the size of the P value. The smaller the P value is, the stronger the difference between groups is.

**TABLE S3 ANOSIM species composition difference analysis results between groups**

| **Method name** | **R statistic** | **P-value** | **Number of permutations** |
| --- | --- | --- | --- |
| ANOSIM | 0.0346 | 0.133 | 999 |

**Note:** In this table, the R value is the statistic of ANOSIM, and the value is between - 1 and 1, representing the difference between intergroup differences and intragroup differences. The closer the R value is to 1, the greater the difference between groups, and the smaller the difference within groups, the better the grouping effect. If R=0, the grouping effect of the sample is equal to the random distribution, and there is no observable statistical difference between the sample groups; If R is negative, the intragroup difference exceeds the intergroup difference, indicating a poor grouping effect. The P value reflects the statistical significance of the ANOSIM analysis results. The smaller the P value is, the higher the significance of the difference between the sample groups.

**TABLE S4: Differential metabolite results**

| **Name** | **RT[Min]** | **FC** | **P value** | **ROC** | **VIP** | **Up or Down** |
| --- | --- | --- | --- | --- | --- | --- |
| Tridecane, 6-methyl- | 35.2649 | 0.447104 | 0.016276 | 0.726496 | 1.258848 | down |
| Furan, 2,3-dihydro-4-methyl- | 9.20617 | 0.637575 | 0.006344 | 0.705128 | 1.48878 | down |
| Acetonitrile | 5.39035 | 4.934623 | 4.30E-05 | 0.92735 | 1.663602 | up |
| Hepten-2-yl tiglate, 6-methyl-5- | 16.3771 | 0.498187 | 0.028405 | 0.709402 | 1.158172 | down |
| Hexane, 2,2,3-trimethyl- | 1.97003 | 5.844466 | 0.017033 | 0.666667 | 1.313802 | up |
| Propanoyl chloride, 3-chloro- | 10.2231 | 7.38956 | 0.035098 | 0.576923 | 1.110062 | up |
| Binapacryl | 22.5441 | 11.63642 | 0.044422 | 0.547009 | 1.845169 | up |
| 6-Hepten-1-ol, 2-methyl- | 19.4715 | 0.51496 | 0.000483 | 0.82906 | 1.404733 | down |
| Butane sultam, N-(3-benzoyloxy-2-butyl)- | 28.8198 | 0.380448 | 0.001218 | 0.833333 | 1.334797 | down |
| Ethyl ether | 1.87877 | 3.610656 | 0.001631 | 0.803419 | 1.52642 | up |
| Dichloroacetic acid, 4-methylpentyl ester | 17.7722 | 0.59545 | 0.042356 | 0.735043 | 1.431924 | down |
| (R)-(-)-2-Pentanol | 9.74073 | 0.546803 | 0.008309 | 0.75641 | 1.294437 | down |
| Cyclohexanol, 5-methyl-2-(1-methylethyl)-, [1S-(1?2?5?]- | 23.8132 | 0.455479 | 0.007917 | 0.722222 | 1.463973 | down |
| 1-Butanol, 3-methyl- | 12.244 | 0.594367 | 0.003448 | 0.782051 | 1.413428 | down |
| 6-Azathymine | 13.813 | 0.278298 | 0.033944 | 0.769231 | 1.190513 | down |
| 2-Butanol | 6.38559 | 0.57939 | 0.01602 | 0.722222 | 1.052093 | down |
| (S)-Dibutyl 3-hydroxybutyl phosphate | 24.2956 | 0.417861 | 0.048877 | 0.777778 | 1.388911 | down |
| 1-Ethyl-3-vinyl-adamantane | 29.5629 | 0.206189 | 0.007321 | 0.816239 | 1.519907 | down |
| Undecane, 2,3-dimethyl- | 10.0928 | 0.481799 | 0.004507 | 0.777778 | 1.317204 | down |
| 2,3-Pentanedione | 7.22003 | 6.778179 | 0.006474 | 0.752137 | 1.439407 | up |
| Dibutyl phthalate | 42.8879 | 0.535804 | 7.24E-05 | 0.905983 | 1.164671 | down |
| 1-(1,3-benzodioxol-5-yl)-N-(1,3-benzodioxol-4-ylmethyl)-N-methylpropan-2-amine | 24.6085 | 0.458897 | 0.007823 | 0.790598 | 2.230182 | down |
| 1-Butanol, 2-methyl- | 12.2745 | 0.331452 | 0.000188 | 0.850427 | 1.89334 | down |
| 3,3-Dimethylglutaric acid | 11.9224 | 0.581297 | 0.007961 | 0.722222 | 1.290447 | down |
| 1-Butanol, 3-methyl-, acetate | 10.8403 | 0.351177 | 0.012261 | 0.735043 | 1.876531 | down |
| 3-Octanone | 13.3957 | 3.92946 | 0.000295 | 0.880342 | 5.429113 | up |
| Acetic acid, hexyl ester | 13.8651 | 0.318365 | 0.002387 | 0.790598 | 2.37587 | down |
| [Acetic acid](javascript:;) | 26.9944 | 0.566782 | 0.010438 | 0.75641 | 1.515639 | down |
| 3-Hexen-1-ol, acetate, (Z)- | 15.2906 | 0.422017 | 0.006061 | 0.782051 | 1.696441 | down |
| 1-Hexanol | 16.5249 | 0.532308 | 0.020069 | 0.769231 | 1.458337 | down |
| 2,4-Cyclohexadien-1-one, 3,5-bis(1,1-dimethylethyl)-4-hydroxy- | 44.6523 | 3.587171 | 0.007534 | 0.786325 | 1.665805 | up |
| Urea, N-(3,4-difluorophenyl)-N'-(3-methyl-1-phenyl-1H-pyrazol-5-yl)- | 27.6246 | 0.258025 | 0.001687 | 0.799145 | 1.996592 | down |
