# Supplementary material for: Intestinal acetic acid regulates the synthesis of sex pheromones in captive giant pandas
Source: Front Microbiol. 2023 Aug 25;14:1234676. doi: 10.3389/fmicb.2023.1234676 (PMC10485365; doi:10.3389/fmicb.2023.1234676)
Supplement: Supplementary file 2 [file Data_Sheet_2.docx]

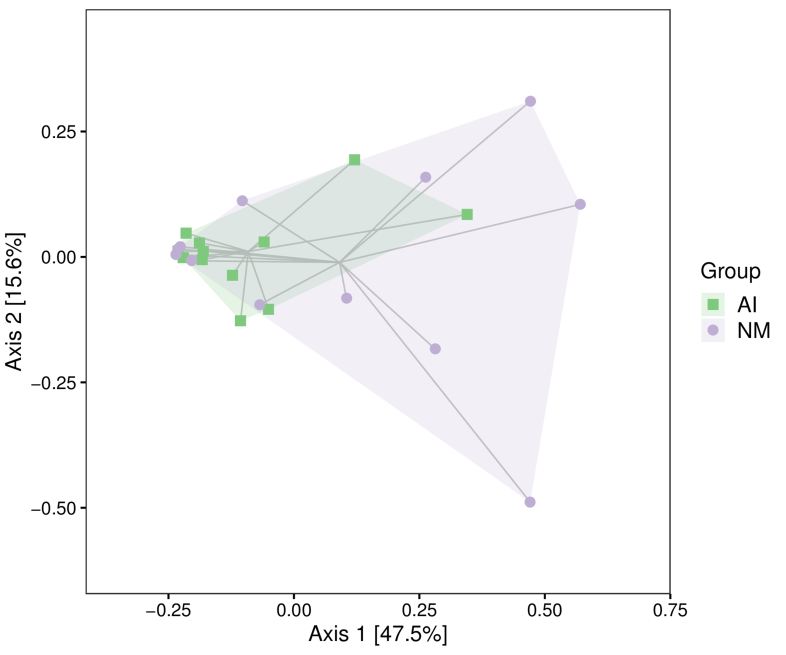


**Fig. S1. PCoA clustering circle plot of the faecal microbiota of giant pandas between the NM and AI groups.**

**Notes：**In this figure, each point represents a sample, and the points with different colours belong to different samples (groups). The closer the distance between the two points, the smaller the difference in the species composition of the two samples and the higher the similarity. The percentages in brackets on the axes represent the proportion of the variance in the raw data that can be explained by the corresponding principal coordinates.


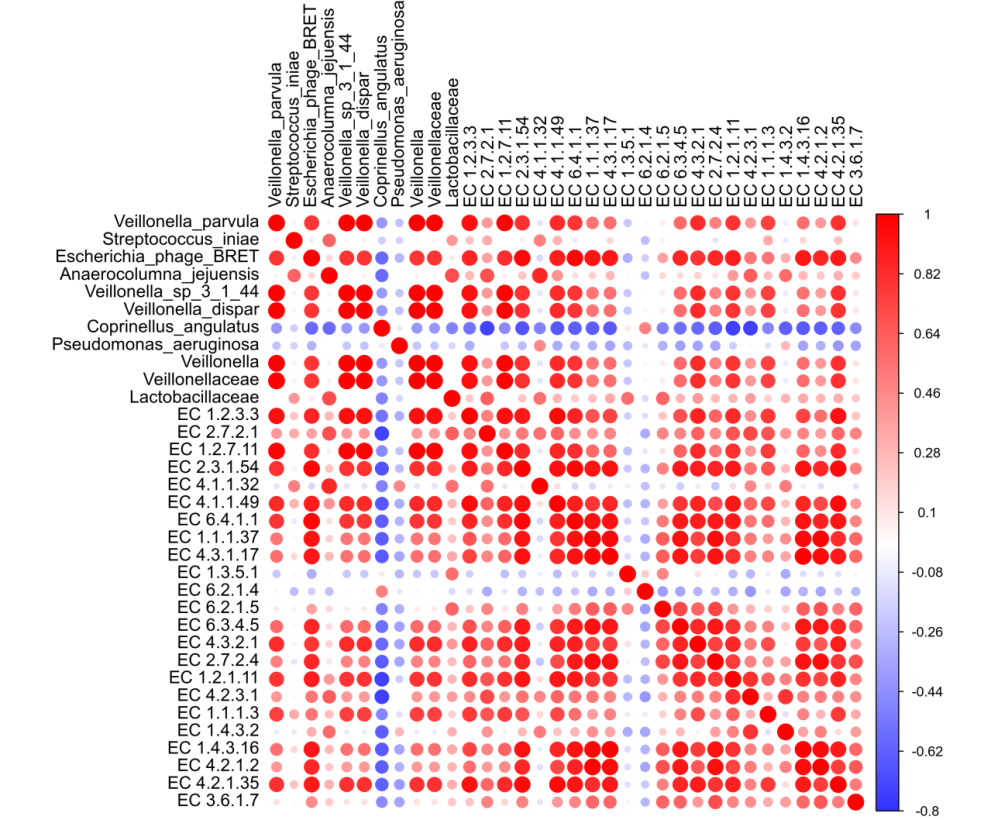


**Fig. S2 Heat map of correlation between significantly different Intestinal microorganisms and functional enzymes.**

**Notes:** Red indicates positive correlation, blue indicates negative correlation, and white indicates non-significant correlation. The color depth is related to the absolute value of the correlation coefficient, that is, the higher the degree of positive or negative correlation, the darker the color. The size of the point is related to the significance of the correlation, the more significant, the smaller the *p*-value and the larger the point is.


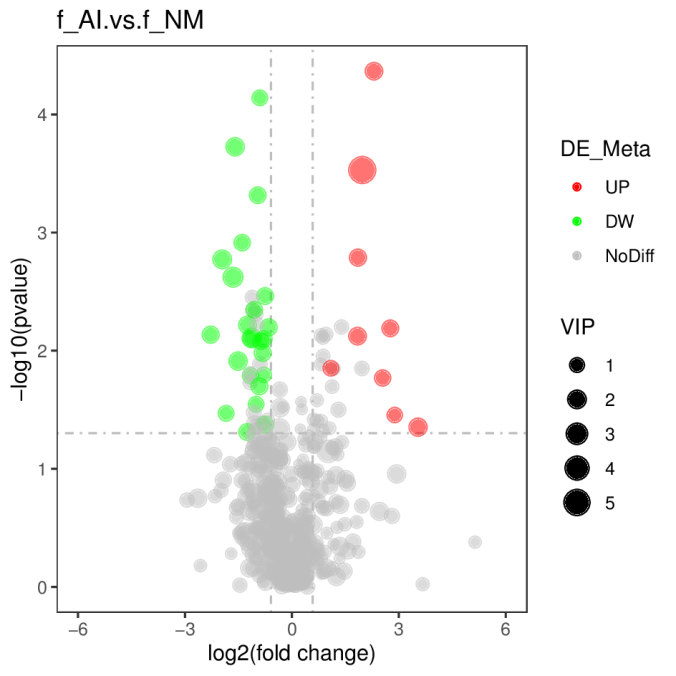


**Fig. S3 Volcano plot of urine metabolites between NM group and AI group.**

**Note:** The abscissa in the figure was the log value of log2 of the Fold Change, and the ordinate was the log value of -log10 of the significance *p* value. Significantly different metabolites: metabolites that meet FC*>* 1.5 and *p* value *<*0.05 were represented in red, and metabolites that meet FC *<*0.67 and *p* value *<*0.05 were represented in blue. Non-significantly different metabolites were shown in black.

**b**

**a**


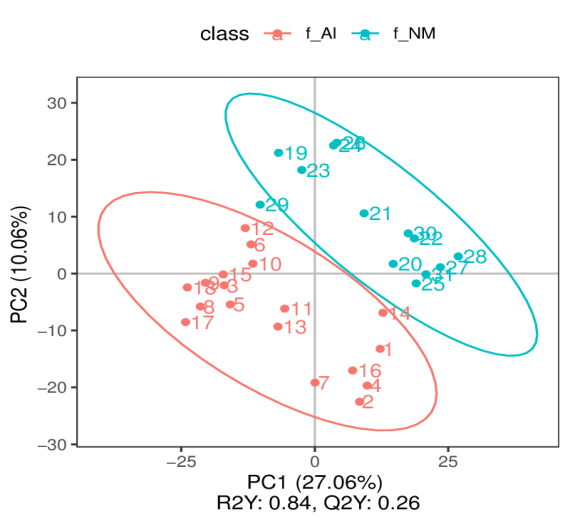

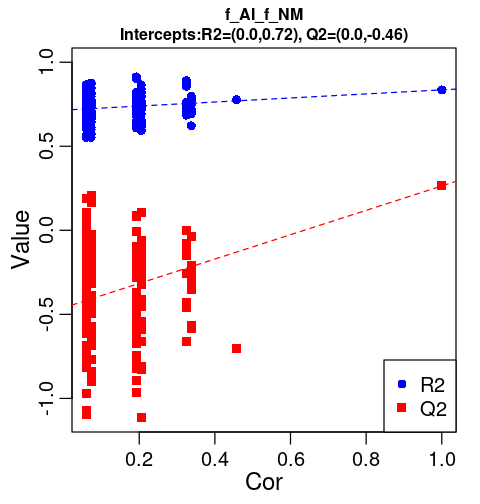


**Fig. S4 PLS-DA Score Scatter Chart (a) and Replacement Test Chart (b)**

**Note: Score Scatter Chart**: The abscissa is the score of the sample on the first principal component; The ordinate is the score of the sample on the second principal component; R2Y represents the interpretation rate of the model, Q2Y is used to evaluate the predictive ability of the PLS-DA model, and when R2Y is greater than Q2Y, it indicates that the model is well established.

**Replacement Test**: The abscissa represents the correlation between the random grouping Y and the original grouping Y, and the ordinate represents the scores of R2 and Q2.


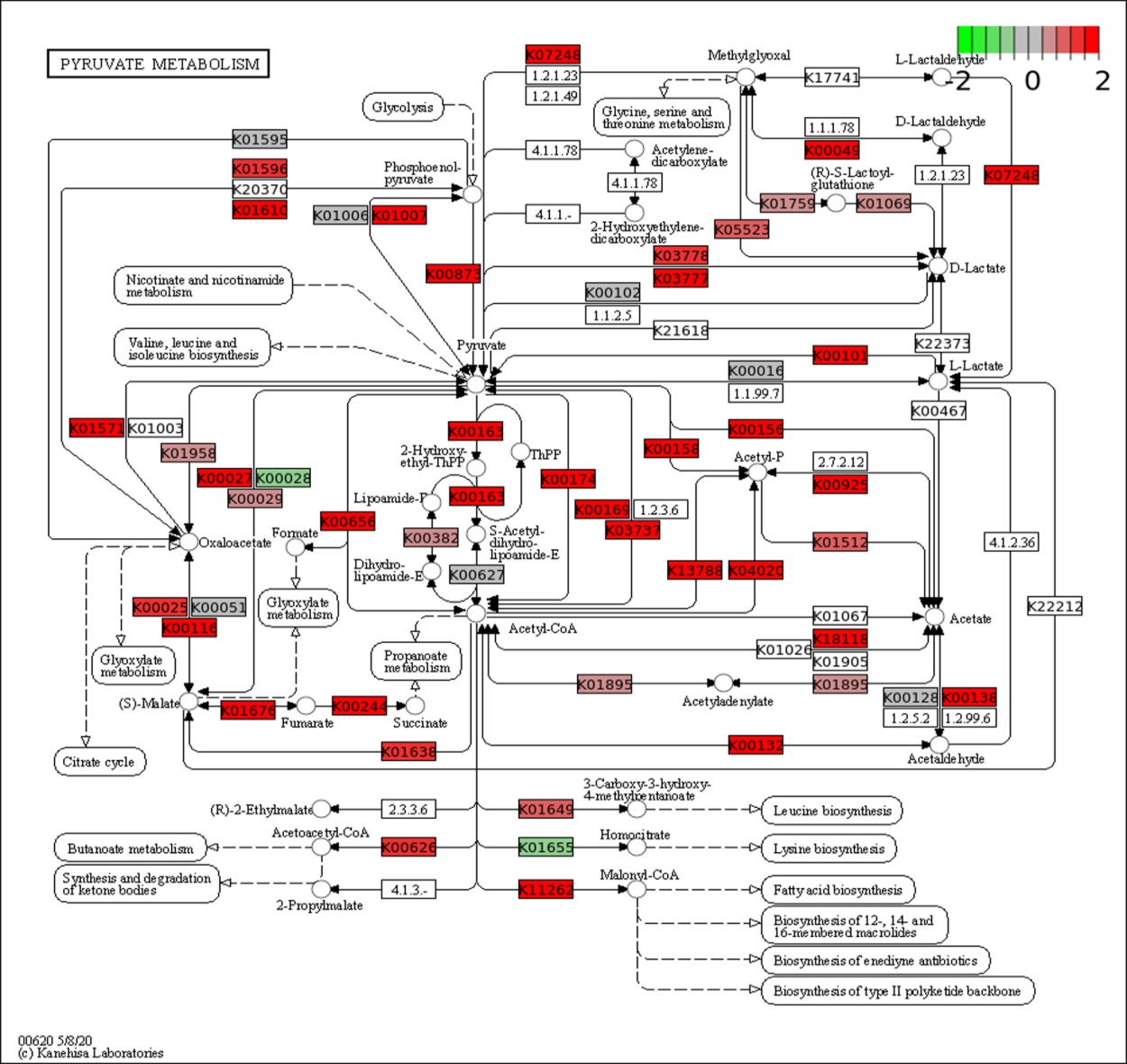


**Fig. S5 Visual analysis of relative abundance of enzymes in pyruvate metabolic pathway (ko00620) in the microbiomes of giant pandas**

**Notes:** Red to green indicates a gradual decrease of relative abundance of enzymes in feces samples, and white indicates these enzymes were not annotated in feces.


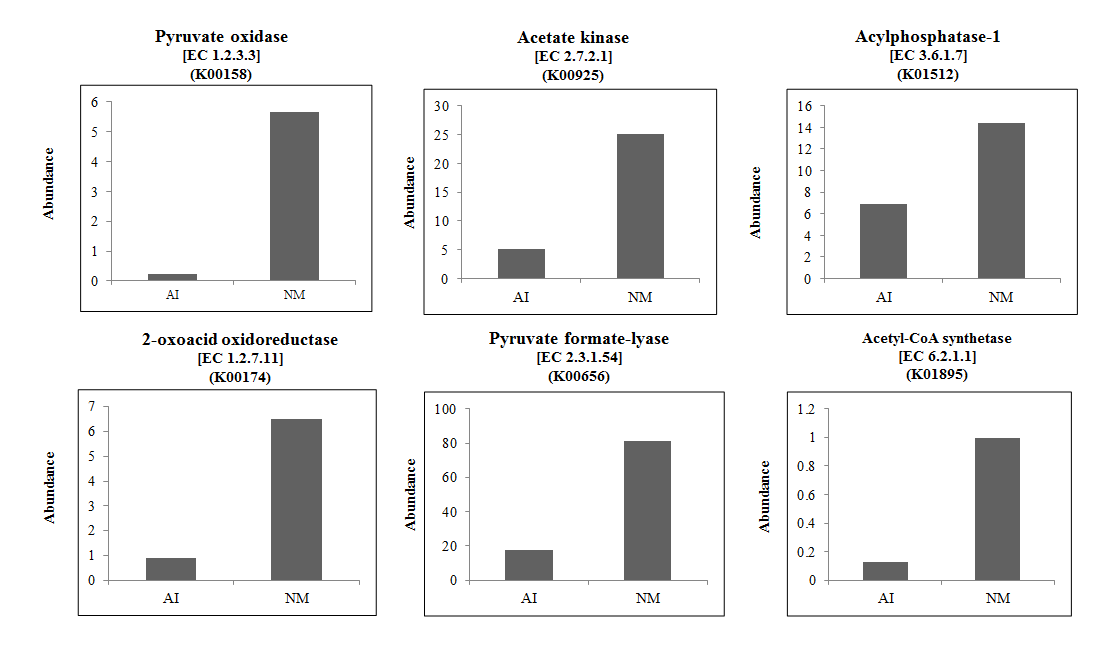


**Fig. S6 Abundance of 4 key proteases represented by pyruvate metabolism pathway (ko00620) in giant panda fecal microbiome**

**NM:** Giant panda with normal expression of natural mating behavior**, AI:** Giant panda with abnormal expression of natural mating behavior

**
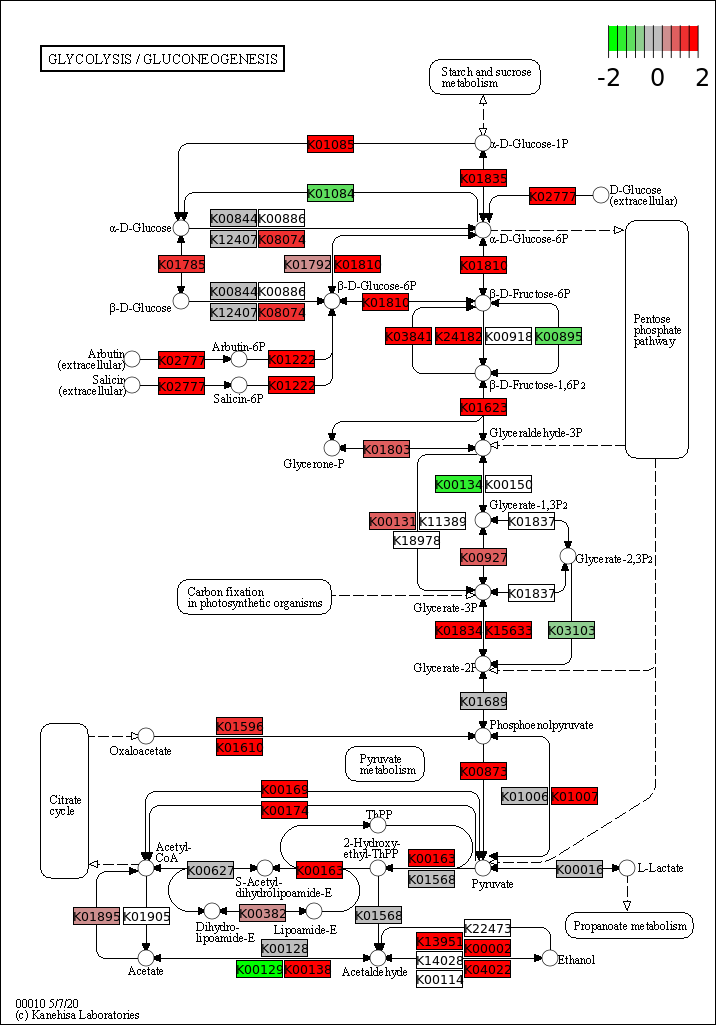
**

**Fig. S7 Visual analysis of relative abundance of enzymes in glycolysis_gluconeogenesis (ko00053) in the microbiomes of giant pandas**

**Notes:** Red to green indicates a gradual decrease of relative abundance of enzymes in feces samples, and white indicates these enzymes were not annotated in feces.


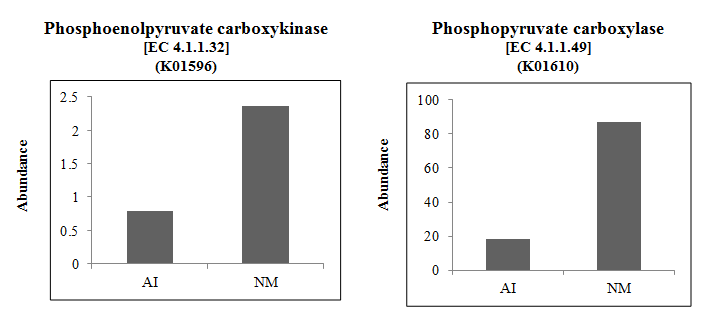


**Fig. S8 Abundance of 2 key proteases represented by glycolysis_gluconeogenesis pathway (ko00010) in giant panda fecal microbiome.**

**NM:** Giant panda with normal expression of natural mating behavior**, AI:** Giant panda with abnormal expression of natural mating behavior


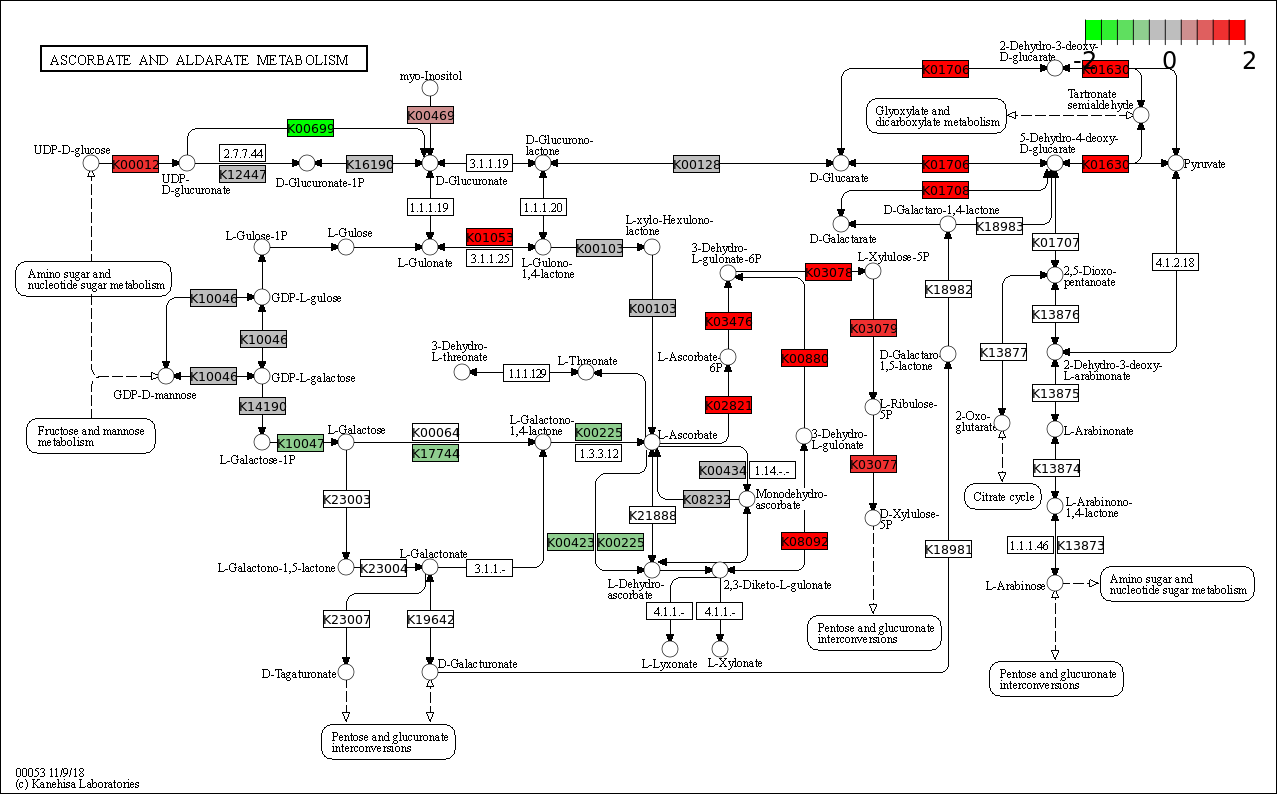


**Fig. S9 Visual analysis of relative abundance of enzymes in ascorbate_and_aldarate_metabolism (ko00053) in the microbiomes of giant pandas**

**Notes:** Red to green indicates a gradual decrease of relative abundance of enzymes in feces samples, and white indicates these enzymes were not annotated in feces.


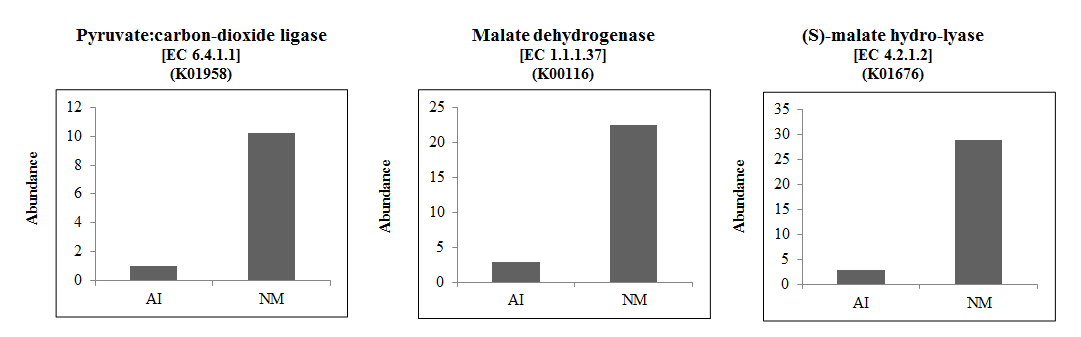


**Fig. S10 Abundance of 3 key proteases represented by ascorbate_and_aldarate_metabolism pathway (ko00053) in giant panda fecal microbiome.**

**NM:** Giant panda with normal expression of natural mating behavior**, AI:** Giant panda with abnormal expression of natural mating behavior

**
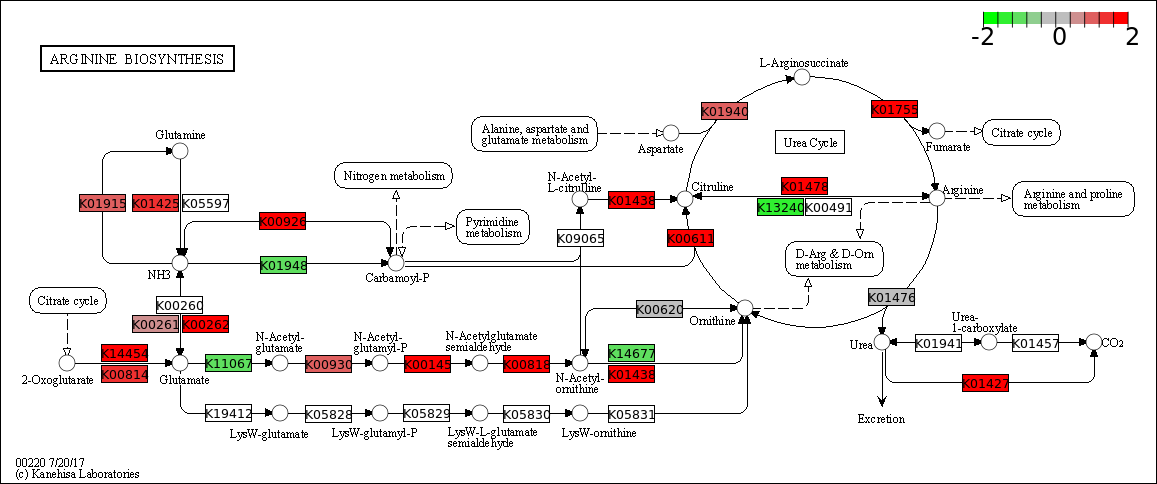
**

**Fig. S11 Visual analysis of relative abundance of enzymes in arginine biosynthesis pathway(ko00220) in the microbiomes of giant pandas**

**Notes:** Red to green indicates a gradual decrease of relative abundance of enzymes in feces samples, and white indicates these enzymes were not annotated in feces.


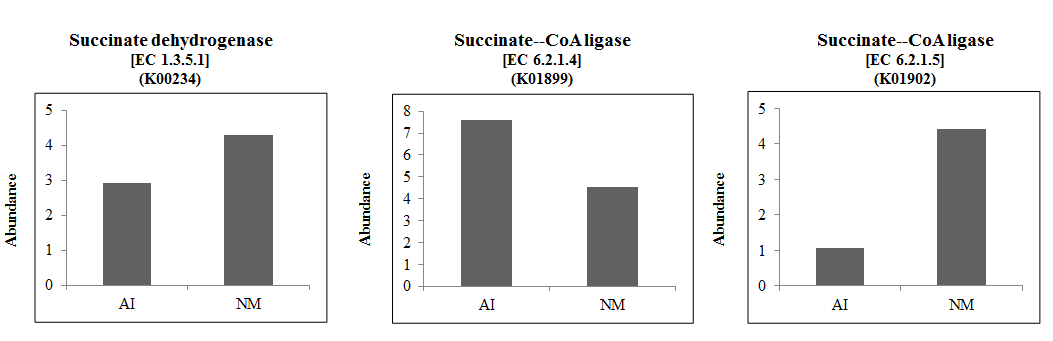


**Fig. S12 Abundance of 2 key proteases represented by arginine biosynthesis pathway (ko00220) in giant panda fecal microbiome.**

**NM:** Giant panda with normal expression of natural mating behavior**, AI:** Giant panda with abnormal expression of natural mating behavior


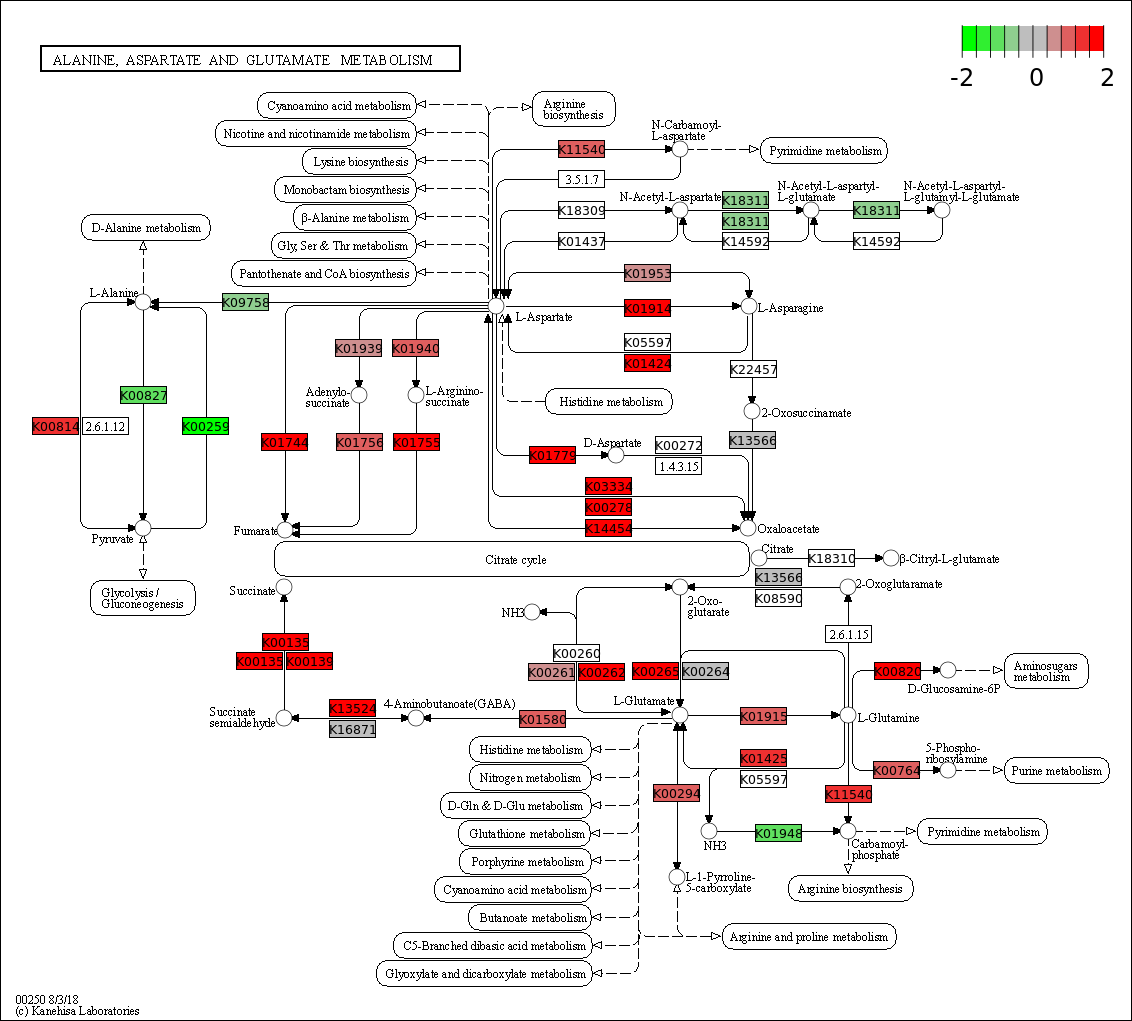


**Fig. S13 Visual analysis of relative abundance of enzymes in alanine_aspartate_and_glutamate_metabolism (ko00250) in the microbiomes of giant pandas**

**Notes:** Red to green indicates a gradual decrease of relative abundance of enzymes in feces samples, and white indicates these enzymes were not annotated in feces.


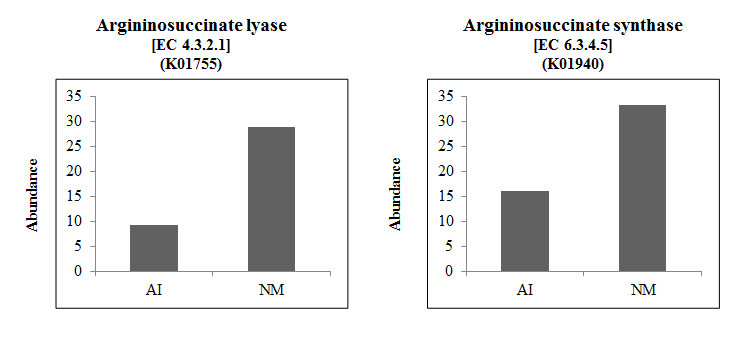


**Fig. S14 Abundance of 3 key proteases represented by alanine_aspartate_and_glutamate_metabolism pathway (ko00250) in giant panda fecal microbiome.**

**NM:** Giant panda with normal expression of natural mating behavior**, AI:** Giant panda with abnormal expression of natural mating behavior


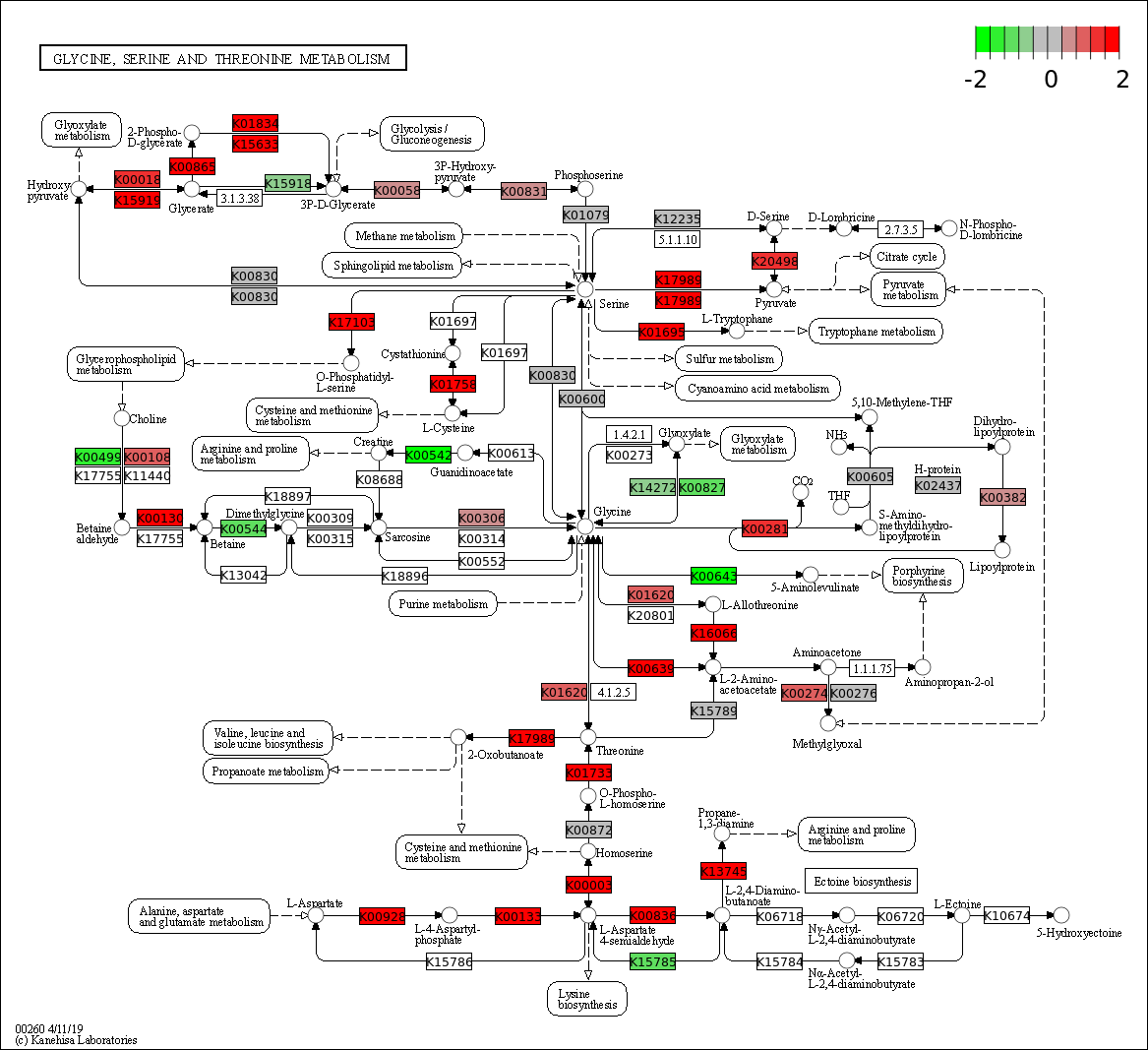


**Fig. S15 Visual analysis of relative abundance of enzymes in glycine_serine_and_threonine_metabolism (ko00260) in the microbiomes of giant pandas**

**Notes:** Red to green indicates a gradual decrease of relative abundance of enzymes in feces samples, and white indicates these enzymes were not annotated in feces.


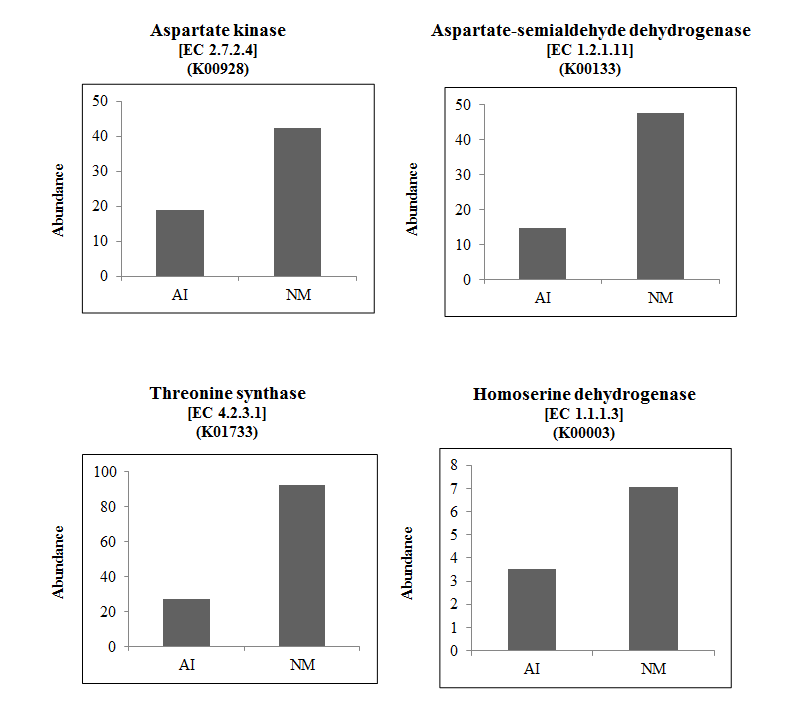


**Fig. S16 Abundance of 4 key proteases represented by glycine_serine_and_threonine_metabolism pathway (ko00220) in giant panda fecal microbiome.**

**NM:** Giant panda with normal expression of natural mating behavior**, AI:** Giant panda with abnormal expression of natural mating behavior


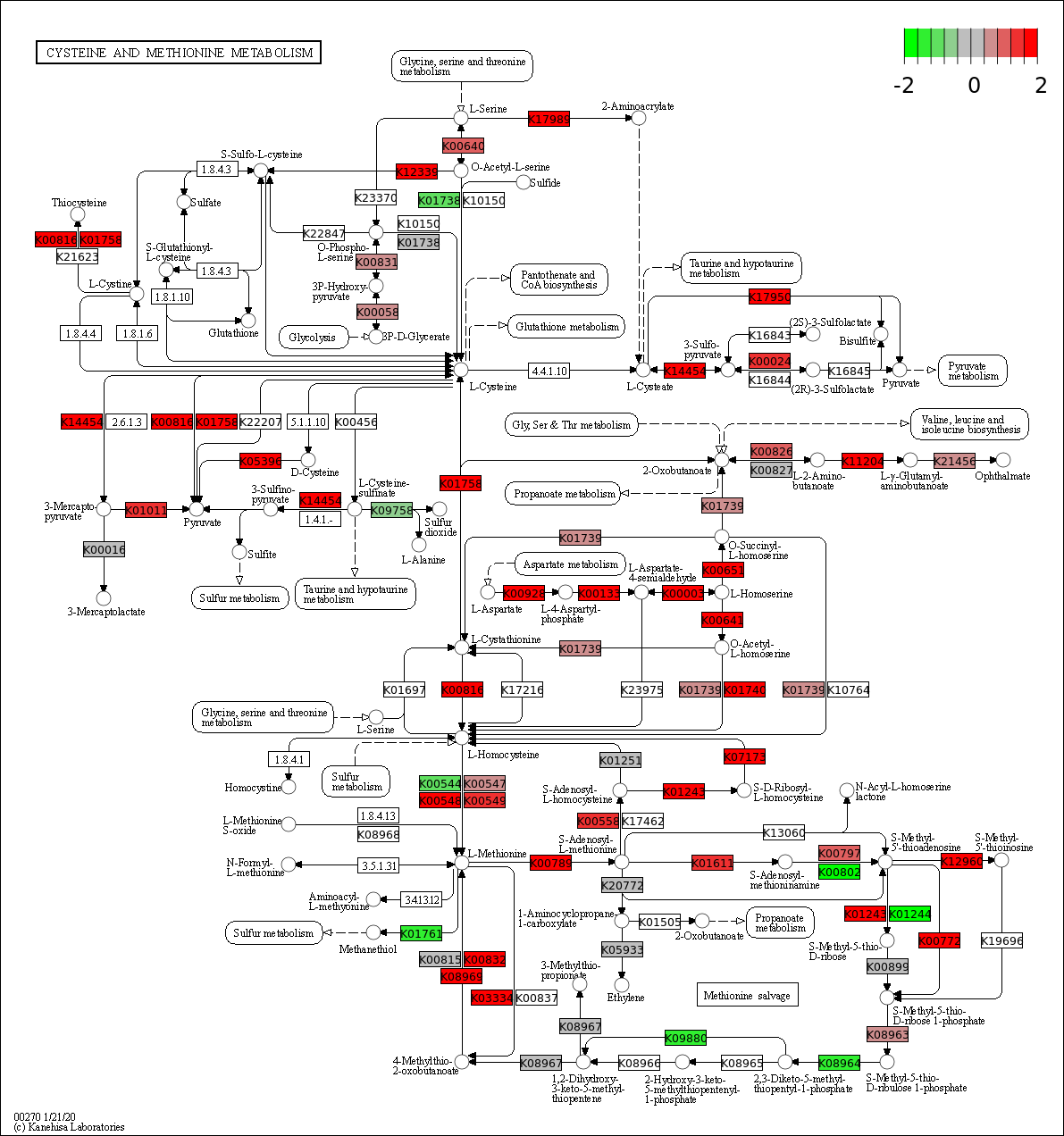


**Fig. S17 Visual analysis of relative abundance of enzymes in cysteine_and_methionine_metabolism (ko00270) in the microbiomes of giant pandas**

**Notes:** Red to green indicates a gradual decrease of relative abundance of enzymes in feces samples, and white indicates these enzymes were not annotated in feces.


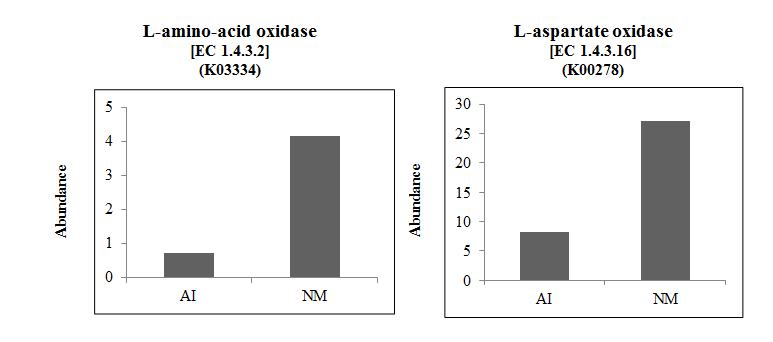


**Fig. S18 Abundance of 2 key proteases represented by cysteine_and_methionine_metabolism pathway (ko00270) in giant panda fecal microbiome.**

**NM:** Giant panda with normal expression of natural mating behavior**, AI:** Giant panda with abnormal expression of natural mating behavior


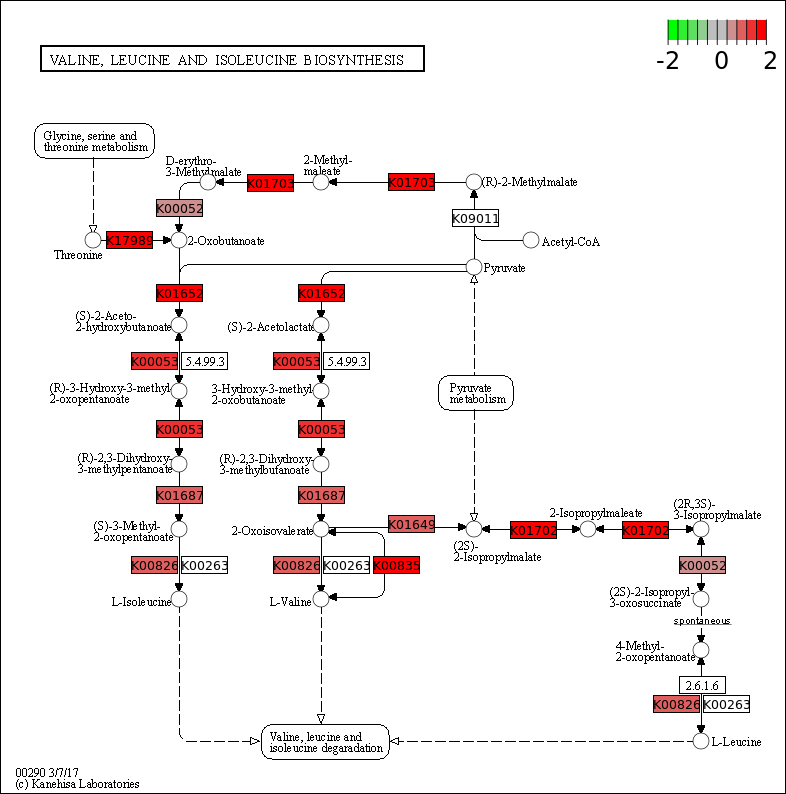


**Fig. S19 Visual analysis of relative abundance of enzymes in valine_leucine_and_isoleucine_biosynthesis (ko00290) in the microbiomes of giant pandas**

**Notes:** Red to green indicates a gradual decrease of relative abundance of enzymes in feces samples, and white indicates these enzymes were not annotated in feces.


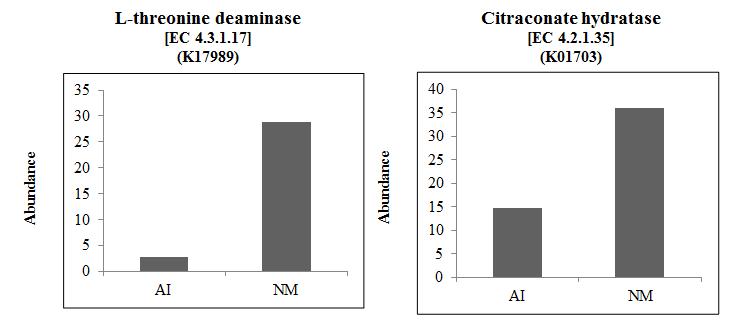


**Fig. S20 Abundance of 2 key proteases represented by valine_leucine_and_isoleucine_biosynthesis pathway (ko00290) in giant panda fecal microbiome.**

**NM:** Giant panda with normal expression of natural mating behavior**, AI:** Giant panda with abnormal expression of natural mating behavior
